# Epidemiology of vancomycin-resistant enterococci in the United Arab Emirates: a retrospective analysis of 12 years of national AMR surveillance data

**DOI:** 10.3389/fpubh.2023.1275778

**Published:** 2023-11-27

**Authors:** Jens Thomsen, Najiba M. Abdulrazzak, Hussain AlRand, The UAE AMR Surveillance Consortium, Godfred Antony Menezes, Carole A. Moubareck, Dean B. Everett, Abiola Senok, Andreas Podbielski

**Affiliations:** ^1^Department of Environmental and Occupational Health and Safety, Abu Dhabi Public Health Center, Abu Dhabi, United Arab Emirates; ^2^Department of Pathology and Infectious Diseases, Khalifa University, Abu Dhabi, United Arab Emirates; ^3^Al Kuwait Hospital Dubai, Emirates Health Services Establishment (EHS), Dubai, United Arab Emirates; ^4^Public Health Sector, Ministry of Health and Prevention, Dubai, United Arab Emirates; ^5^Department of Medical Microbiology and Immunology, Ras Al Khaimah (RAK) Medical and Health Sciences University, Ras Al Khaimah, United Arab Emirates; ^6^College of Natural and Health Sciences, Zayed University, Dubai, United Arab Emirates; ^7^Research Center, Khalifa University, Abu Dhabi, United Arab Emirates; ^8^Infection Research Unit, Khalifa University, Abu Dhabi, United Arab Emirates; ^9^College of Medicine, Mohammed Bin Rashid University of Medicine and Health Sciences, Dubai, United Arab Emirates; ^10^School of Dentistry, Cardiff University, Cardiff, United Kingdom; ^11^Institute of Medical Microbiology, Virology and Hygiene, University Medicine, Rostock, Germany

**Keywords:** vancomycin-resistant enterococci (VRE), vancomycin, antimicrobial resistance (AMR), United Arab Emirates (UAE), surveillance

## Abstract

**Introduction:**

Enterococci are usually low pathogenic, but can cause invasive disease under certain circumstances, including urinary tract infections, bacteremia, endocarditis, and meningitis, and are associated with peritonitis and intra-abdominal abscesses. Increasing resistance of enterococci to glycopeptides and fluoroquinolones, and high-level resistance to aminoglycosides is a concern. National antimicrobial resistance (AMR) surveillance data for enterococci from the Middle East and North Africa (MENA) and the Gulf region is scarce.

**Methods:**

A retrospective 12-year analysis of *N* = 37,909 non-duplicate diagnostic *Enterococcus* spp. isolates from the United Arab Emirates (UAE) was conducted. Data was generated by routine patient care during 2010–2021, collected by trained personnel and reported by participating surveillance sites to the UAE National AMR Surveillance program. Data analysis was conducted with WHONET.

**Results:**

*Enterococcus faecalis* was the most commonly reported species (81.5%), followed by *Enterococcus faecium* (8.5%), and other enterococci species (4.8%). Phenotypically vancomycin-resistant enterococci (VRE) were found in 1.8% of *Enterococcus* spp. isolates. Prevalence of VRE (%VRE) was highest for *E. faecium* (8.1%), followed by *E. faecalis* (0.9%). A significant level of resistance to glycopeptides (%VRE) for these two species has been observed in the majority of observed years [*E. faecalis* (0–2.2%), 2010: 0%, 2021: 0.6%] and *E. faecium* (0–14.2%, 2010: 0%, 2021: 5.8%). Resistance to fluoroquinolones was between 17 and 29% (*E. faecalis*) and was higher for *E. faecium* (between 42 and 83%). VRE were associated with higher patient mortality (RR: 2.97), admission to intensive care units (RR: 2.25), and increased length of stay (six excess inpatient days per VRE case), as compared to vancomycin-susceptible *Enterococcus* spp.

**Discussion:**

Published data on *Enterococcus* infections, in particular VRE-infections, in the UAE and MENA region is scarce. Our data demonstrates that VRE-enterococci are relatively rare in the UAE, however showing an increasing resistance trend for several clinically important antibiotic classes, causing a concern for the treatment of serious infections caused by enterococci. This study also demonstrates that VRE were associated with higher mortality, increased intensive care unit admission rates, and longer hospitalization, thus poorer clinical outcome and higher associated costs in the UAE. We recommend the expansion of current surveillance techniques (e.g., local VRE screening), stricter infection prevention and control strategies, and better stewardship interventions. Further studies on the molecular epidemiology of enterococci are needed.

## 1 Introduction

Several dozen species of enterococci are part of the physiological intestinal flora in humans as well as in vertebrate and invertebrate animals (1). Due to a high degree of tenacity, once excreted, the bacteria stay viable or may even proliferate on environmental surfaces, food as well as in surface and waste water (2–8). The bacteria are transmitted between humans and from animals to humans by hand contact as well as by contaminated food and water (9, 10).

In addition to their physiologic role in the human intestinal microbiome, they can cause infections, especially in persons with breaches in their unspecific immune defense, e.g., due to inserted catheters, surgical procedures and medication affecting the mucosal surfaces (11–14). In such persons, enterococci as sole responsible agents can cause urinary tract infections, bacteremia, and endocarditis. In combination with other, more pathogenic bacteria they are associated with wound infections and secondary peritonitis (15–21).

Once causing infections, antibiotic therapy can be challenging, since enterococci are inherently resistant to cephalosporins and often also to penicillins (22–24). So, in severe infections, glyco- and lipopeptides such as vancomycin and daptomycin, or oxazolidinones such as linezolid are among the few remaining therapeutic options (25, 26). But even to these compounds, enterococci have developed resistance mechanisms encoded on mobile genetic elements or plasmids (27, 28). So far, this type of vancomycin resistance encoded by the *vanA* or *vanB* genes has predominantly been demonstrated in *Enterococcus faecium* but may also be present in *Enterococcus faecalis* (29–33).

There is conflicting data on the role of vancomycin-resistant enterococci (VRE) in severe infections concerning their contribution to increased mortality (34–37). However, there are potentially more tenacious and/or pathogenic VRE clones which remain for extended periods in specific hospitals and as a consequence, are involved in nosocomial outbreaks (38–42) combined with a high economic burden (43–45).

Therefore, important national and international institutions such as the United States Centers for Disease Prevention and Control (CDC) (46) and the European Center for Disease Prevention and Control (ECDC) (47) have included VRE on their lists of potentially harmful microorganisms that should be constantly monitored.

Data from such monitoring programs indicate that the VRE portion among the total number of clinical enterococcal isolates varies between 1 and 50% depending on regional and temporal settings and also across individual medical institutions within a given region and period. Preventive hygiene measures such as contact precautions and isolation of VRE-carrying/infected patients are not necessarily associated with changed VRE portions among enterococci, stressing the importance of individual VRE clones for the regional and temporal VRE prevalence (48–51).

Increasing levels of antimicrobial resistance in healthcare and non-healthcare settings is also increasingly seen as a problem in the Middle East and North African (MENA) region, including the Gulf region (GCC, Gulf Cooperation Council) (52, 53). Several reports from countries belonging to the MENA and GCC region demonstrate the emergence of and increasing interest in VRE. These countries include Morocco (54), Algeria (55–57), Tunisia (58, 59), Libya (60), Egypt (61–66), Saudi-Arabia (67–69), Oman (70), Qatar (71), Bahrain (72), Iran (73), and others. However, published epidemiological data from the MENA region on *Enterococcus* spp. and VRE on a national/country level are scarce and outdated, and, to the best of our knowledge, limited to Saudi Arabia (69) and Oman (70).

Surveillance of antimicrobial resistance in the United Arab Emirates (UAE) started in 2010 at Emirate-level (Abu Dhabi). Inspired by the World Health Organization (WHO) global action plan on antimicrobial resistance (GAP-AMR) and especially, the global AMR surveillance system (GLASS), the UAE national antibiotic resistance surveillance program was established in 2015, leading to the present data collection and evaluation.

Here we present the enterococci epidemiology in the UAE in a period ranging from pre-COVID-19 pandemic years to well into the second pandemic year (2021). The successful impact of the UAE health care system on the relatively low VRE prevalence, as well as the impact of VRE on the UAE health care system and health outcomes are discussed. This paper also presents a discussion of the effect that the COVID-19 pandemic had on the surveillance and reporting of *Enterococcus* spp., and related antimicrobial resistance levels during the pre-pandemic and pandemic period. This paper represents the first documentation of a 12-year resistance portfolio for enterococci across the whole country, from 2010 until 2021.

## 2 Materials and methods

### 2.1 Study design and data source

A multi-institutional retrospective observational study was conducted between 2010 and 2021 in the UAE using data extracted from the WHONET microbiology laboratory database software (https://whonet.org/) supported by the Global AMR Surveillance System protocol (GLASS, World Health Organization). Data was generated, collected, cleaned and analyzed through the UAE national AMR Surveillance programs as described by Thomsen et al. (74).

### 2.2 Identification and enrollment of national AMR surveillance sites

Starting in 2010, UAE institutions were incorporated into the UAE national AMR surveillance program based on epidemiological needs assessment, readiness and willingness of facilities to participate, availability of high-quality electronic AMR data, lab accreditation status, and qualification of staff. Hospitals, centers, and clinics representing all seven Emirates of the UAE joined the AMR surveillance network gradually over the years.

### 2.3 Bacterial population and variables of the study

All *Enterococcus* spp. isolated from clinical samples at the National AMR surveillance sites from January 2010 to December 2021 were included in this study. Only the first reported isolate per patient was included in the surveillance analysis.

The associated patient demographic information, clinical data, and microbiologic laboratory results were extracted from the national WHONET laboratory database software. The demographic variables included age, sex, nationality, clinical variables revealed the type of facility reporting the isolate (hospital/center/clinic), patient location, location type, specimen collection date, types of infection/specimen source, and microbiology variables revealed types of organism and antibiotic susceptibility testing results. The infection was considered as community-acquired if the patient presented at an outpatient setting (center, clinic), emergency department or urgent care center, or a clinic or outpatient department of a hospital. The infection was considered healthcare-associated if the isolate was reported from an inpatient setting (inpatient ward, ICU).

### 2.4 Bacterial identification

The participating centers used at least one commercial, automated system for identification of bacteria, including VITEK^®^ (BioMérieux SA, Craponne, France), BD Phoenix™ (Becton Dickinson, New Jersey, USA) and MicroScan WalkAway (Beckman Coulter, Brea, CA, USA). Only one lab relied on manual systems like API^®^ (Analytical Profile Index. BioMérieux SA, Craponne, France) solely for identification.

### 2.5 Antimicrobial susceptibility testing

Antimicrobial susceptibility testing was performed at the National AMR surveillance sites using at least one commercial, automated system for routine antimicrobial susceptibility testing. Only two laboratories used manual testing methods (disc diffusion/Kirby Bauer). All laboratories followed Clinical & Laboratory Standards Institute (CLSI) guidelines for antimicrobial susceptibility testing (75). The criteria of the susceptibility of tigecycline were adapted from the European Committee on Antimicrobial Susceptibility Testing (EUCAST) guidelines (76). Any *Enterococcus* spp. phenotypically resistant to either vancomycin, or teicoplanin, or both, was considered as vancomycin-resistant *Enterococcus* spp. (VRE). To assess the multidrug-resistant (MDR) phenotype of the isolates the standard definition by Magiorakos et al. (77) was used. To assess the extensively drug-resistant (XDR) and pandrug-resistant (PDR) phenotypes, a slightly modified version of the standard definition by Magiorakos et al. (77) was used. Magiorakos' et al. definitions for XDR and PDR phenotypes for *Enterococcus* spp. includes 11 antimicrobial categories with 17 antibiotic agents. For technical reasons, associated costs, and local formulary requirements, participating laboratories would not routinely test all 17 antibiotics, i.e., some antibiotics were only very rarely (minocycline, meropenem) or not at all (doripenem) tested.

As such, the following, slightly modified definitions were used for “possible XDR” and “possible PDR” isolates (modifications highlighted in *italics*):

- **“Possible XDR”**: Non-susceptibility to at least one agent *routinely tested by clinical labs* in all but two or fewer antimicrobial categories (i.e., bacterial isolates remain susceptible to only one or two categories).
- **“Possible PDR”**: Non-susceptibility to all agents *routinely tested by clinical labs* in all antimicrobial categories (i.e., no agents were tested as susceptible for that organism).

### 2.6 Statistical tests

Significant temporal trends for antimicrobial resistance were assessed if at least five years of data were available to perform such an analysis. Trend analysis was not done when <30 isolates per year were reported. Extended Mantel-Haenszel Chi-square test for trend was done using SPSS version 29.0.1.0. Statistically significant differences in mortality among patients admitted in the intensive care unit (ICU) were assessed and *p* < 0.05 was considered significant. To assess differences in the length of stay between those patients with and without VRE, we performed a weighted log-rank test, and a *p* < 0.05 was considered statistically significant.

## 3 Results

### 3.1 Distribution of reporting sites for national AMR surveillance

**Figure 1:**
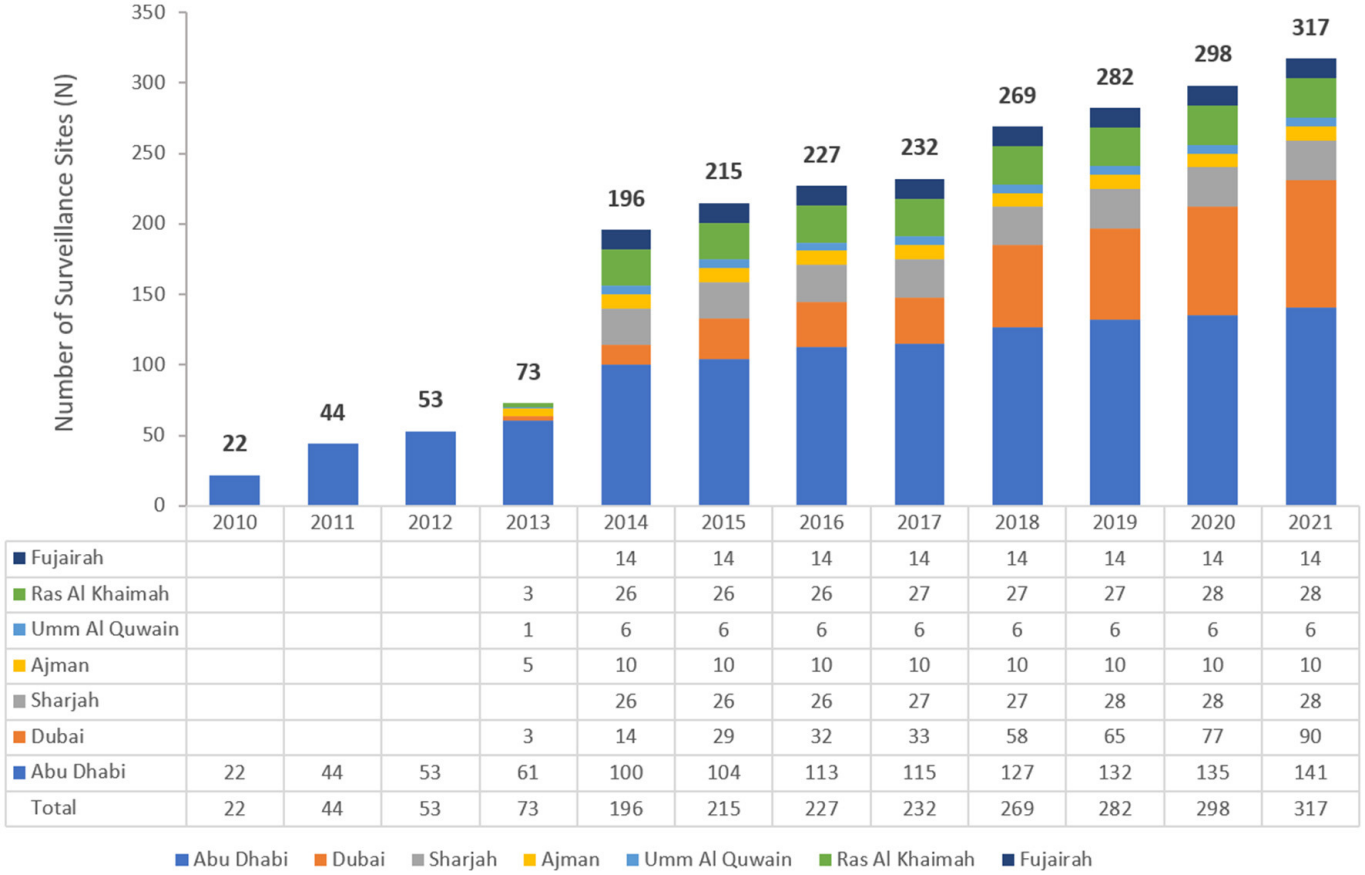
Number of surveillance sites participating in National AMR surveillance over the surveillance period (2010–2021), by year and Emirate.

The UAE national AMR surveillance program was initiated in 2010 in the Abu Dhabi Emirate with 6 hospitals and 16 centers/clinics enrolled. Additional sites were recruited over the years, starting with 22 participating sites located only in the Emirate of Abu Dhabi in 2010, which is the first year during which the study was initiated, and reaching in 2021 a total of 317 surveillance sites, including 84 hospitals and 233 centers/clinics and representing all seven Emirates of the country. Figure 1 represents the distribution of reporting sites by Emirate from 2010 to 2021.

### 3.2 Bacterial population

**Figure 2:**
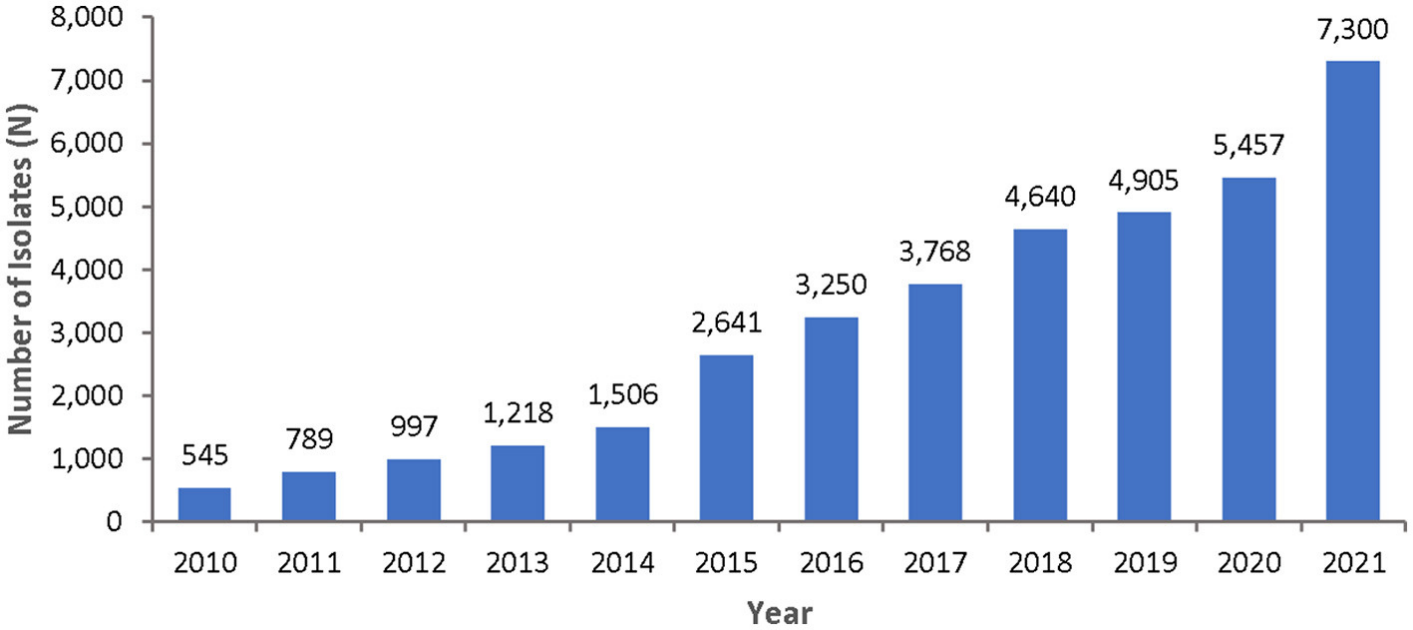
Numbers of non-repetitive *Enterococcus* spp. isolated per year over the surveillance period (2010–2021), by year.

From 2010 to 2021, a total of 37,909 non-repetitive *Enterococcus* spp. were isolated from an equivalent number of patients over the surveillance period. Figure 2 represents the number of *Enterococcus* spp. included per year.

### 3.3 Species distribution

**Figure 3:**
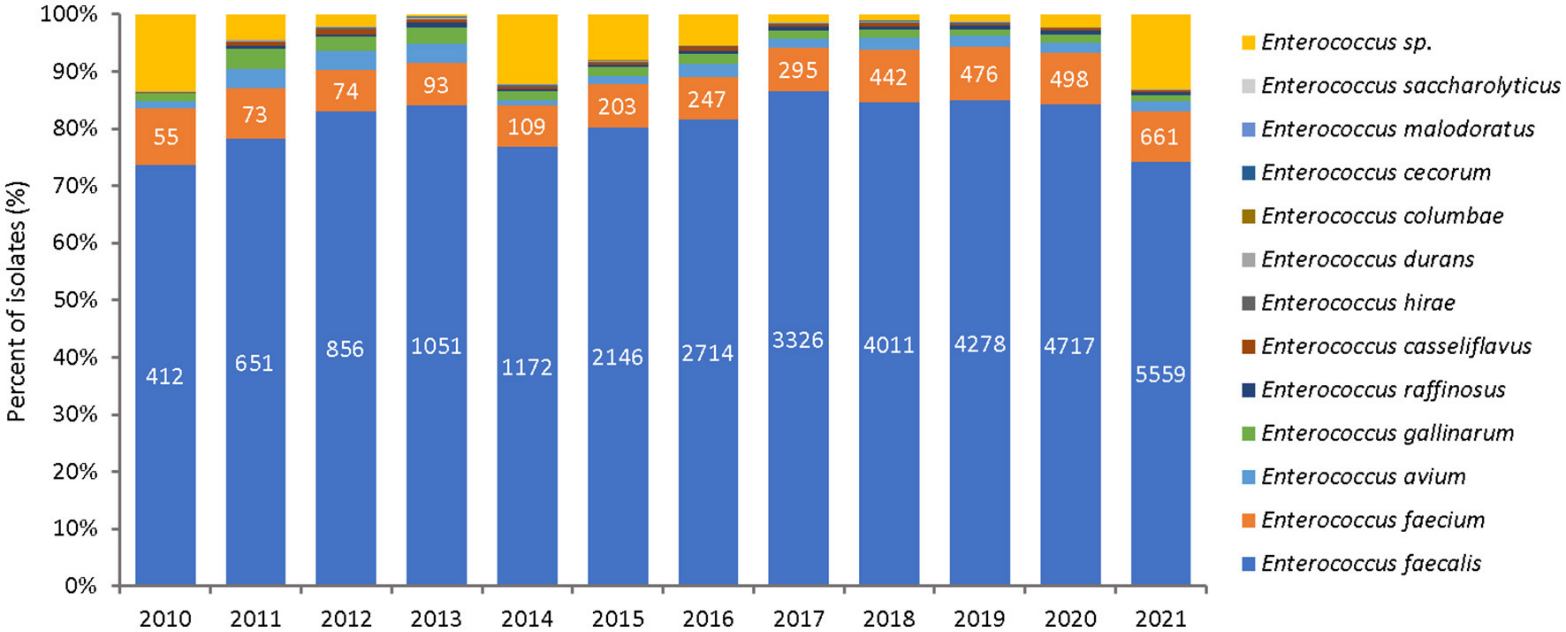
Species distribution of *Enterococcus* spp. over the surveillance period (2010–2021), by year and species.

Among the 37,909 *Enterococcus* spp. analyzed, *E. faecalis* was the most commonly reported species (81.5%), followed by *E. faecium* (8.5%), and other enterococci species (4.8%). The species distribution over the surveillance period is shown in Figure 3 and the overall percentages over the study period are shown in Supplementary Table 1.

### 3.4 Distribution of *Enterococcus* spp. patients by age, gender, nationality status, and emirate

**Figure 4:**
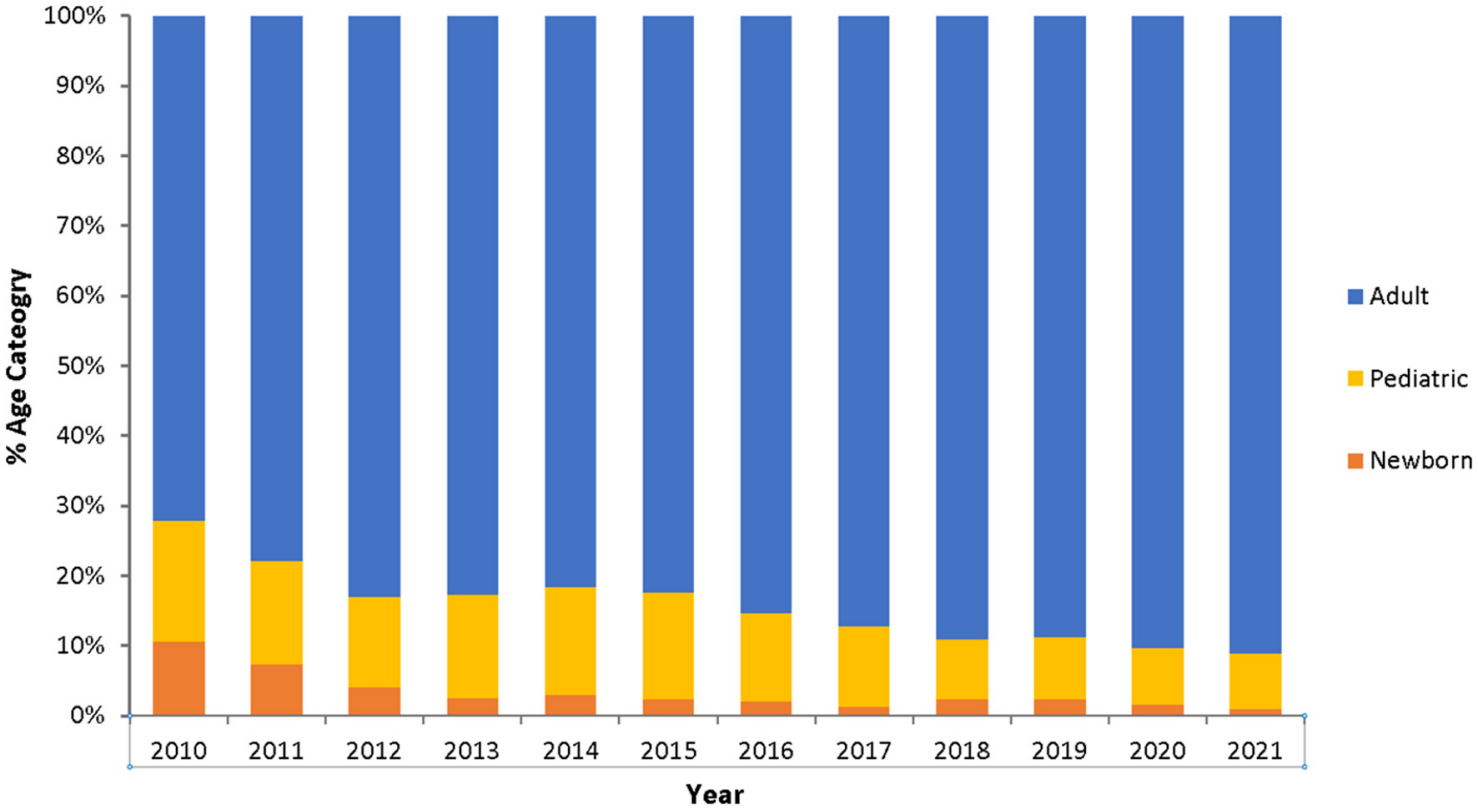
Age distribution of *Enterococcus* spp. patients over the surveillance period (2010–2021), by year and age category. Newborn: 0–30 days, Pediatric: 1 month to 18 years, Adult: 19+ years.

*Enterococcus* spp. strains were mostly associated with adults (Figure 4). It is noteworthy that the proportion of inpatient and outpatient surveillance sites changed during 2010–2021. While in 2010 inpatient sites accounted for 67.5% of all reported isolates of *Enterococcus* spp., this percentage decreased to 31.8% in 2021, due to the enrollment of more outpatient sites over time, as compared to inpatient sites. Accordingly, during the same period (2010–2021), the percentage of *Enterococcus* spp. isolates from outpatient sites increased from 31.7% (2010) to 56.1% (2021). As all newborn and most pediatric samples likely originate from several inpatient sites, a “decrease” of percentage of infections in the newborn and pediatric population over time is observed, however this is a statistical artifact due to the change in proportions of sites over time.

**Figure 5:**
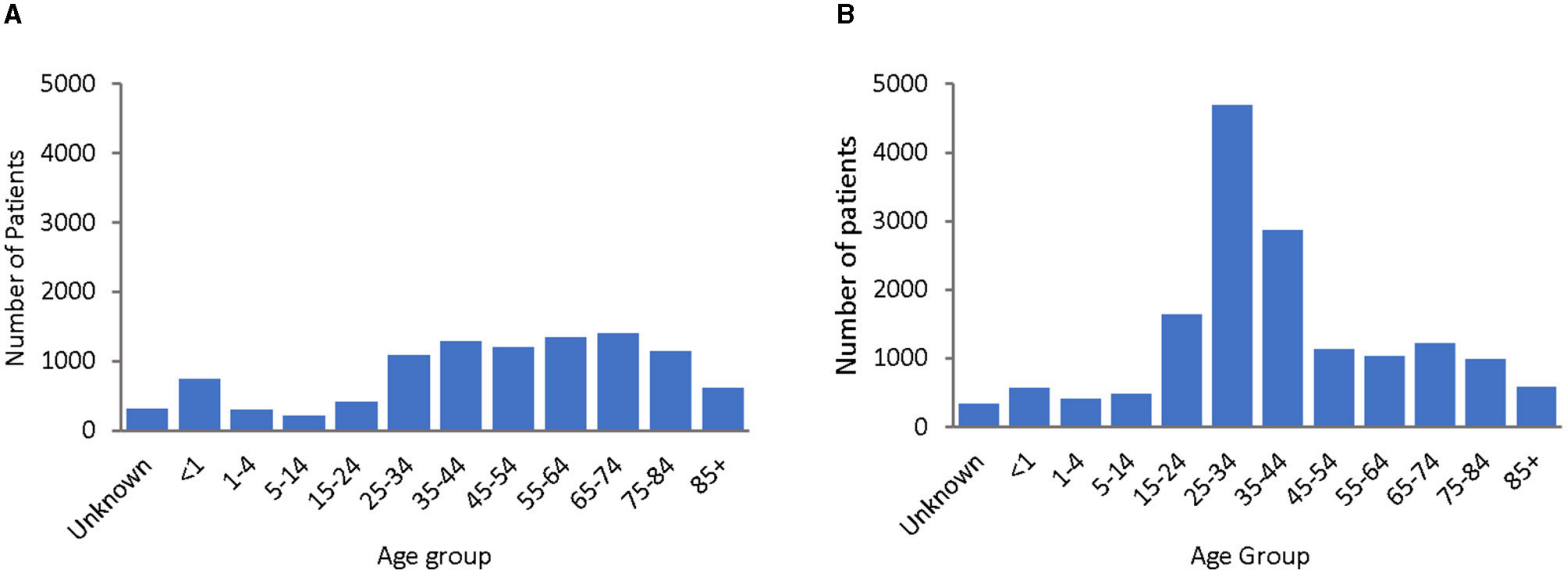
Gender and age distribution of *Enterococcus* spp. patients over the surveillance period (2010–2021), by male **(A)** and female **(B)** gender and age group.

*Enterococcus* spp. was more commonly found in females (61.2%), as compared to males (38.8%), with a predominance of younger females (age 15–44), which was not observed in the male patient population (Figure 5).

Among those patients for whom the nationality status was available (*n* = 21,975, 59.7%), 41.5% of these patients were UAE nationals, while 58.5% were expatriates. For the remaining 40.3% of patients the nationality status was missing. Non-nationals were from a total of 136 countries, most commonly from Asian and Arab countries (India, 8.7%; Pakistan, 6.7%; Egypt, 4.6%; Yemen, 3.7%; Syria, 3.6%, Jordan, 3.1%, others, 27.9%).

### 3.5 Distribution of *Enterococcus* spp. by sample type group

**Figure 6:**
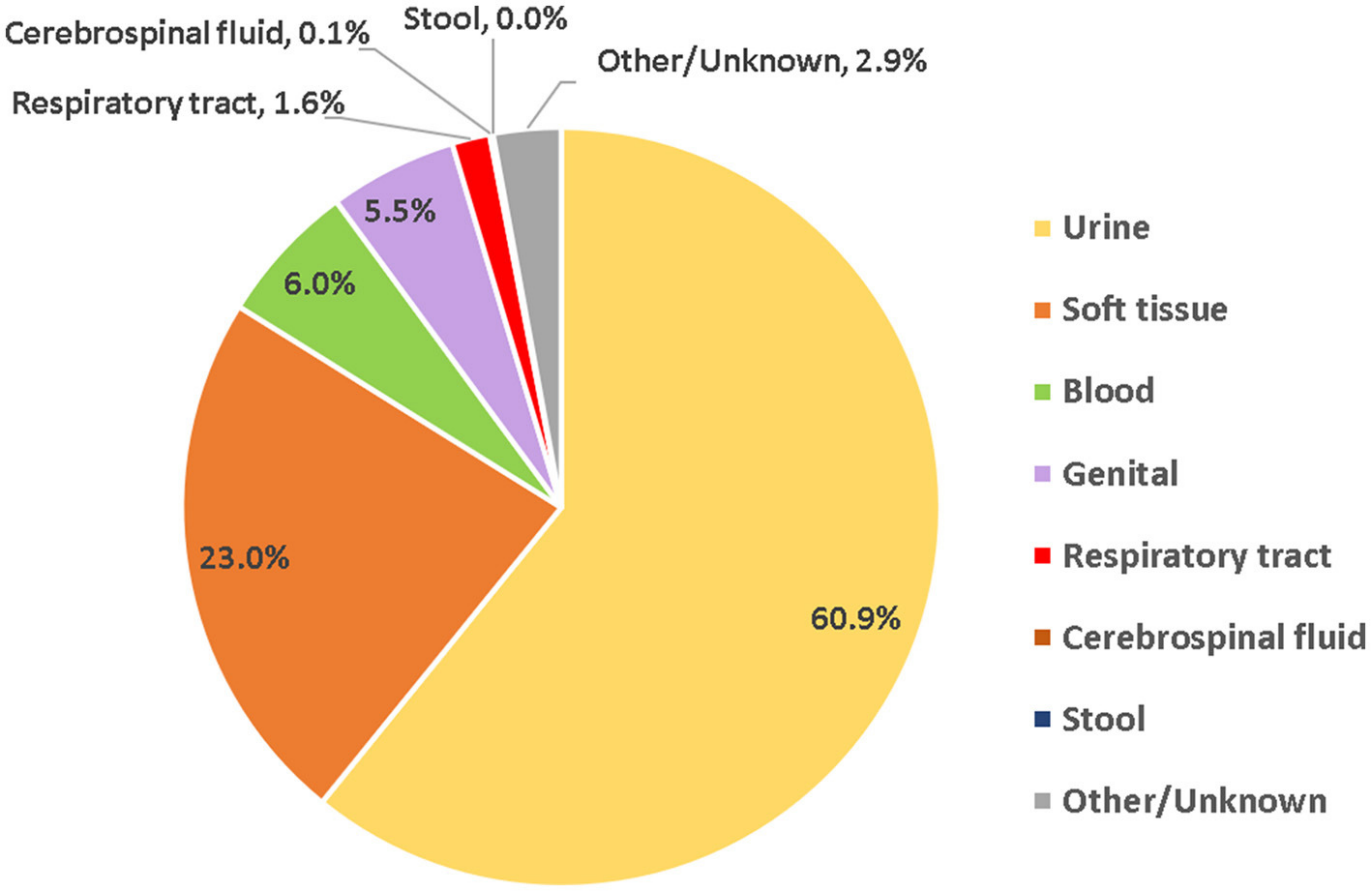
Distribution of *Enterococcus* spp. non-duplicate isolates/patients over the surveillance period (2010–2021), by sample type group.

Most of the *Enterococcus* spp. strains were isolated from urine (60.9%), followed by soft tissue (23.0%, including wound swabs: 5.5%), blood (6.0%), and genital (5.5%, including vaginal swabs: 4.6%), and other groups. The distribution of *Enterococcus* spp. isolates by clinical sample type is shown in Figure 6.

### 3.6 Distribution of *Enterococcus* spp. by location type (inpatients/outpatients/ICU), and department

*Enterococcus* spp. isolates/patients were primarily detected in community settings (outpatient clinics and emergency wards, 54.0%), whereas 46.0% were found in inpatient settings (including ICU: 10.7%).

By clinical specialty/department, *Enterococcus* spp. isolates/patients were associated with internal medicine (17.9%), obstetrics and gynecology (14.9%), surgery (12.7%), and various other disciplines (32.9%). For the remaining 21.6% the department was not known.

### 3.7 Trend of antimicrobial susceptibility profiles of *Enterococcus* spp.

**Figure 7:**
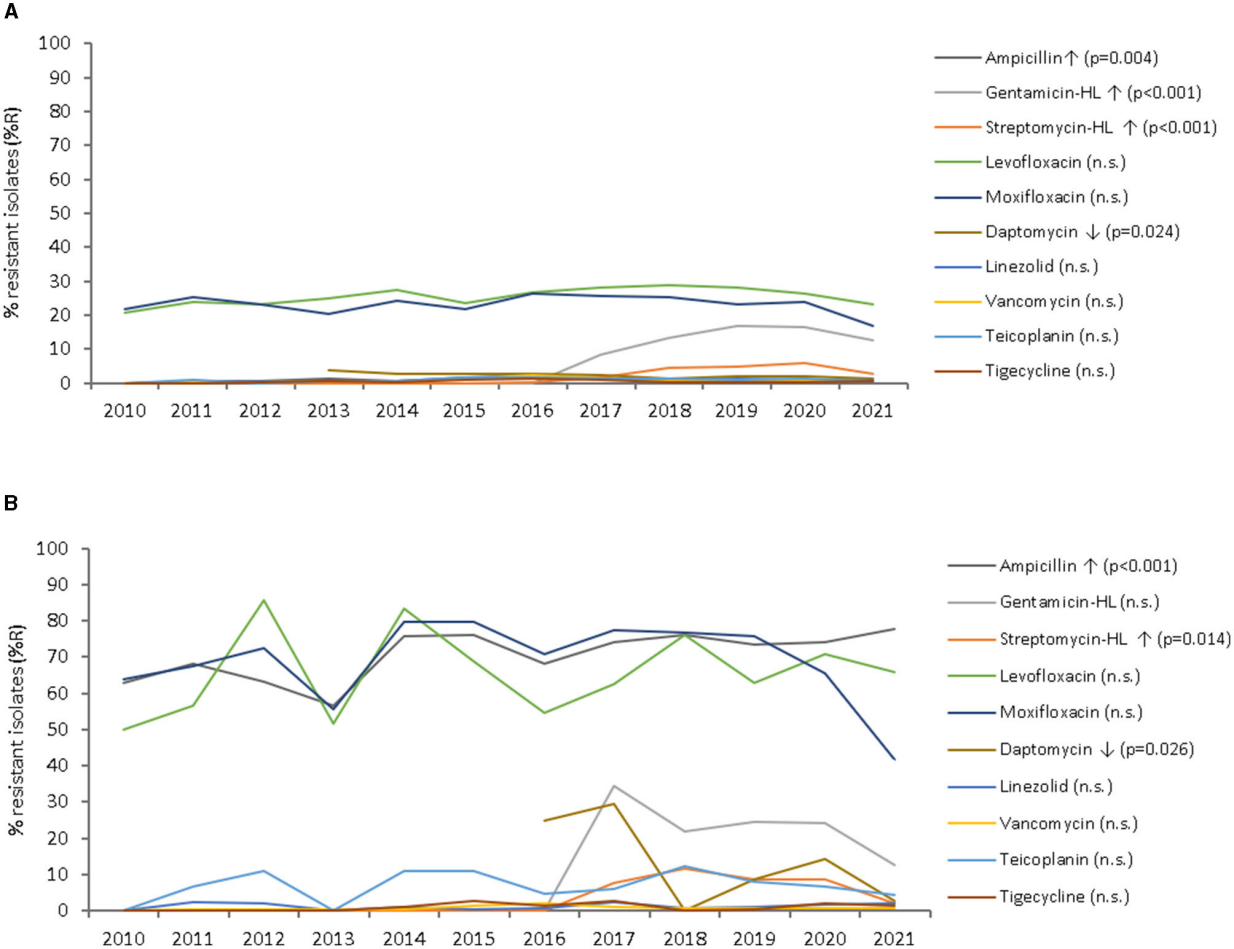
Resistance trends of *Enterococcus faecalis* **(A)** and *Enterococcus faecium* **(B)** to 10 antibiotics over the period of the study (2010–2021), by year and antibiotic.

The trend of antimicrobial sensitivity of all *Enterococcus* spp. recovered during the period of the study (2010 to 2021) is shown in Figure 7.

As shown in Figure 7, *E. faecium* showed an overall higher level of antimicrobial resistance during the study period (2010–2021), as compared to *E. faecalis*; in particular for aminopenicillins (ampicillin), fluoroquinolones (levofloxacin, moxifloxacin), aminoglycosides (gentamicin-HL, streptomycin-HL), and glycopeptides (vancomycin, teicoplanin).

Resistance to **aminopenicillins** (ampicillin) ranged from 0–1.4% (*E. faecalis*, average: 0.8%) to 63.0%−77.7% (*E. faecium*, average: 70.5%). An increasing trend of resistance to ampicillin was observed for *E. faecalis* (from 0% in 2010 to 0.4% in 2021), and for *E. faecium* (from 63.0% in 2010 to 77.7% in 2021; *p* < 0.001).

Resistance to fluoroquinolones (levofloxacin, moxifloxacin) was in the range of 20%−28% (*E. faecalis*, average: 24.2%) to 42%−83% (*E. faecium*, average: 67.3%), showing a largely horizontal trend (n.s.). Susceptibility to fluoroquinolones was in the range of 68%−78% (*E. faecalis*, average: 72.3%) and 7%−45% (*E. faecium*, average: 25.6%) during the study period.

Resistance to high-level **aminoglycosides** (gentamicin-HL, streptomycin-HL) has not been observed in the UAE during the early years of AMR surveillance (2010–2015), however has emerged since then, with current (2021) levels at 12.5% and 2.5% (*E. faecalis*), and 12.6% and 2.2% (*E. faecium*), respectively. A statistically significant overall increase of resistance to streptomycin-HL has been observed for *E. faecalis*, from 0% (2012) to 2.5% (2021; *p* < 0.001), as well as for *E. faecium*, from 0% (2013) to 2.2% (2021), with a peak of 11.5% in 2018 (*p* = 0.014). Resistance to gentamicin-HL increased from 0% (2013) to 12.5% (2021) for *E. faecalis* (*p* < 0.001), and from 0% (2013) to 12.6% (2021), with a peak of 34.5% (2017) for *E. faecium* (n.s.).

Resistance levels to **glycopeptides** (vancomycin, teicoplanin) were very low for *E. faecalis* (0–2.2%, average: 0.9%), however as high as 0–14.2% (average: 8.1%) for *E. faecium*, with both antibiotics showing a slightly increasing trend over the study period (2010–2021) for both pathogens (statistically not significant, n.s.). Across all *Enterococcus* species, 1.5% of isolates were fully resistant to both, vancomycin and teicoplanin, 0.7% of isolates were resistant to vancomycin and susceptible to teicoplanin, while 97.4% of isolates were fully susceptible to both (co-susceptibility). For *E. faecalis*, 0.8% of isolates were fully resistant to both, vancomycin and teicoplanin (probably *vanA* phenotype), 0.4% of isolates were resistant to vancomycin and susceptible to teicoplanin (probably *vanB* phenotype), while 98.5% of isolates were fully susceptible to both (co-susceptibility). For *E. faecium*, 6.2% of isolates were fully resistant to both, vancomycin and teicoplanin (probably *vanA* phenotype), 1.9% of isolates were resistant to vancomycin and susceptible to teicoplanin (probably *vanB* phenotype), while 91.6% of isolates were fully susceptible to both (co-susceptibility).

Resistance data for **lipopeptides** (i.e., daptomycin) has been available since 2013 for *E. faecalis* and since 2016 for *E. faecium*. Both organisms have shown a decline in resistance to daptomycin. For *E. faecalis*, there was a significant decline in antimicrobial resistance from 3.8 to 1.4% between 2013 and 2021 (*p* = 0.024), and for *E. faecium* from 25.0 to 2.6% between 2016 and 2021 (*p* = 0.026).

Both **linezolid** and **tigecycline** remained highly susceptible over the study period for both pathogens (0–2.8 %R, 94–100 %S).

The impact of the Coronavirus Disease 2019 (COVID-19) pandemic on incidence of multidrug-resistant infections and antimicrobial resistance levels and trends has been subject to scientific debate (78–82). Supplementary Figure 2 shows the number of non-duplicate isolates/patients reported to the national AMR surveillance system during the pre-pandemic period (2010–2019), as compared to the COVID-19 pandemic period (2020–2021). Results are presented for (a) all organisms (A), and (b) all *Enterococcus* spp. isolates/patients (B). The number of reported isolates (all organisms, A) increased during the pre-pandemic period (2010–2019) consistently, from 11,698 (2010) to 105,096 (2019), in line with the increasing number of surveillance sites being enrolled into the program during this pre-pandemic period. For 2020, this number then decreased to *n* = 95,502, and increased again to an all-time high (*n* = 130,750) in 2021, reflecting a short-term negative impact of COVID-19 on national AMR surveillance reporting. The number of isolates reported for *Enterococcus* spp. (B) increased consistently during the whole study period (2010–2021), suggestive of only a minor negative impact of COVID-19 on reporting rates for *Enterococcus* spp., including VRE.

As shown in Figure 7 and Table 1, the percentage of *E. faecalis* and *E. faecium* isolates resistant to antibiotics (%R) was lower, or did not further increase, for most antibiotics (with few exceptions) during the early years of the Coronavirus Disease 2019 (COVID-19) pandemic (2020 and 2021), as compared to the average resistance level during the pre-pandemic period (2010–2019). Resistance to glycopeptides was reduced by 0.3–0.4 (*E. faecalis*) and 2.7 (*E. faecium*) percentage points during COVID-19, as compared to the pre-COVID period. Similarly, resistance to moxifloxacin was reduced by 4.1 (*E. faecalis*) and 19.1 (*E. faecium*) percentage points during COVID-19, as compared to the pre-COVID period, whereas levofloxacin showed a mixed pattern. For daptomycin, resistance was reduced by 0.3 (*E. faecalis*) and 5.9 (*E. faecium*) percentage points during COVID-19, as compared to the pre-COVID period.

**Table 1:** Percentage of *Enterococcus faecalis* and *Enterococcus faecium* isolates resistant to antibiotics (%R), during the pre-COVID-19 pandemic period (2010–2019), and the early COVID-19 pandemic period (2020–2021).

**Organism**	**Antibiotic**	%R pre-COVID-19 (2010–2019)^*^	%R during COVID-19 (2020–2021)^*^	**Difference (%R)**
*Enterococcus faecalis*	Ampicillin	1.0	0.6	−0.4
	Gentamicin-HL	10.2	13.9	3.7
	Streptomycin-HL	2.2	4.2	2.0
	Levofloxacin	26.7	24.4	−2.3
	Moxifloxacin	23.6	19.5	−4.1
	Daptomycin	2.0	1.7	−0.3
	Linezolid	1.3	0.9	−0.4
	Vancomycin	1.0	0.6	−0.4
	Teicoplanin	1.2	0.9	−0.3
*Enterococcus faecium*	Ampicillin	71.8	76.7	4.9
	Gentamicin-HL	19.9	19.5	−0.4
	Streptomycin-HL	5.9	5.5	−0.4
	Levofloxacin	65.6	67.9	2.3
	Moxifloxacin	73.6	54.5	−19.1
	Daptomycin	13.6	7.7	−5.9
	Linezolid	1.1	2.0	0.9
	Vancomycin	9.1	6.4	−2.7
	Teicoplanin	7.6	4.9	−2.7

* %R: weighted average across the respective period.

### 3.8 Trend of MDR, XDR, and PDR phenotypical resistance profiles of *Enterococcus* spp.

**Figure 8:**
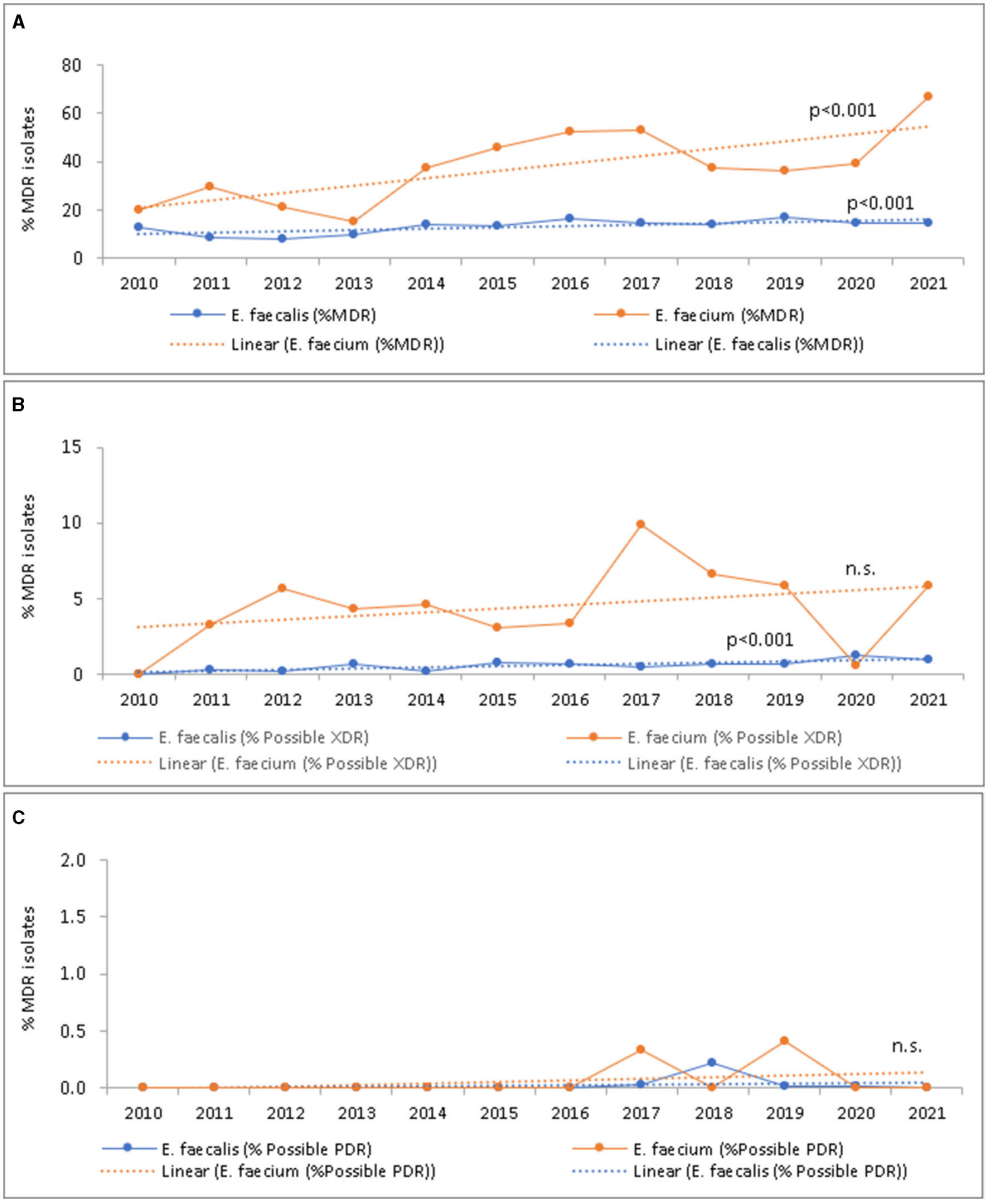
*Enterococcus* species: trend of percent MDR (% MDR) **(A)**, % possible XDR **(B)**, and % possible PDR **(C)** over the study period (2010–2021), by year.

The overall percentage of *E. faecalis* and *E. faecium* isolates that exhibited a multidrug-resistant (%MDR) phenotype, possibly extensively resistant (%possible-XDR), and possibly pandrug-resistant (% possible-PDR) phenotype over the study period is shown in Table 2, whereas Figure 8 presents the trends of such phenotypes over the study period. Overall, multi-, extensively-, and pandrug-resistant phenotypes were more frequently found in *E. faecium* (MDR: 42.7%, possible-XDR: 11.3%, possible PDR: 0.3%), as compared to *E. faecalis* (MDR: 13.9%, possible-XDR: 1.0%, possible PDR: 0.04%; Table 2). As shown in Figure 8, an increasing trend of %MDR and %possible-XDR isolates over the study period has been observed for *E. faecium*, and for *E. faecalis*. For *E. faecium*, %MDR increased from 20.0% (2010) to 66.6% (2021; *p* < 0.001), and % possible-XDR increased from 0% (2010) to 5.9% (2021; n.s.). *Enterococcus faecalis* showed an increasing trend for % possible-XDR, from 0% (2010) to 0.4% (2021; *p* < 0.001).

**Table 2:** *Enterococcus* species: percent MDR (% MDR), % possible XDR, and % possible PDR, as an average over the study period (2010–2021).

**Organism**	**Isolates (*N*)**	**MDR**	**Possible XDR**	**Possible PDR**
*Enterococcus faecalis*	30,893	4,287 (13.9%)	307 (1.0%)	12 (0%)
*Enterococcus faecium*	3,226	1,376 (42.7%)	365 (11.3%)	11 (0%)

### 3.9 Mortality rate

A subgroup analysis including the nine clinical institutions that reported mortality was performed. In these institutions, a total of 12,372 patients were associated with *Enterococcus* spp. (non-VRE) of whom 787 patients died (mortality rate: 6.4%), while a total of 127 patients were associated with *Enterococcus* spp. (VRE), of whom 24 patients died (mortality rate: 18.9%). The difference in mortality between VRE patients (18.9%) and non-VRE patients (6.4%) is statistically highly significant (RR 2.97, 95% CI 2.06, 4.29, *p* < 0.001).

### 3.10 Admission to intensive care unit

A total of 27,839 patients were associated with *Enterococcus* spp. (non-VRE) of whom 2,854 patients were admitted to ICU (ICU admission rate: 10.3%), while a total of 430 patients were associated with *Enterococcus* spp. (VRE), of whom 99 patients were admitted to ICU (ICU admission rate: 23.0%). The difference in ICU admission rate is statistically highly significant (RR 2.25, 95% CI 1.88, 2.69, *p* < 0.001).

### 3.11 Length of stay

A subgroup analysis including those patients for whom the date of admission as well as the date of discharge was known was performed. For those patients who were associated with non-VRE *Enterococcus* spp. (*n* = 3,824) the median length of stay was 7 days, while for those patients who were associated with VRE *Enterococcus* spp. (*n* = 715) the median length of stay was 13 days (Supplementary Figure 3). The weighted log-rank test was done to assess the difference in length of stay (LOS) between patients infected with VRE and those infected with non-VRE. The data showed that there was a statistically significant difference in the length of stay between the two groups, Chi square 5.8, *p* = 0.02 (Supplementary Figure 4).

Based on a total of *n* = 687 patients with infections associated with VRE during the observation period (2010–2021), a total of 4,122 excess days of hospitalization were observed, attributable to VRE. For the year 2021 only, a total of 732 excess hospitalization days were observed, attributable to VRE.

## 4 Discussion

This is the first comprehensive analysis across the UAE that shows their relative significance and magnitude of *Enterococcus* spp. infections in clinical settings, their evolution of antimicrobial resistance over time, and the association of VRE-enterococci with a negative health outcome. The present research utilized an extensive dataset collected over a considerable duration allowing precise observation of subtle variations in antimicrobial resistance among enterococci. This level of inclusive analysis has not been previously replicated in the country. The samples analyzed in this study consisted of non-repetitive enterococcal isolates of laboratory-confirmed identity and antibiotic resistance profile, indicating authenticity of the microbiological material used and accuracy of the generated data.

The UAE accommodates a diverse community comprising more than 200 nationalities, out of which 136 are represented in this study population. Emirati nationals make up approximately 10% of the overall population, highlighting the UAE's status as one of the countries with a significant expatriate presence. Among the expatriate groups in the UAE, Indians and Pakistanis represent the largest segments, accounting for 27.5 and 12.7% of the total population, respectively (83). However, our results show that about 41.5% of *Enterococcus* samples were recovered from Emirati nationals, while the other 58.5% were expatriates. This can partially be explained with the higher rate of healthcare utilization and more comprehensive health insurance coverage among Emirati nationals.

In our study, among expatriate groups, also Indians and Pakistanis represent the largest segments, accounting for 6.1 and 4.5% of the study population. These proportions of the total sample pool should be interpreted cautiously, since 40.3% of the samples attributed from patients for whom their nationality was not coded in the data, hence not available. With the expatriate-inclusive and multicultural setting expected to prevail for the forthcoming years, the UAE may be an interesting niche to compare how trends of resistance in enterococci differ by nationality, shedding a light on cultural and social factors contributing to resistance in a multidisciplinary research perspective, as previously suggested (84, 85). However, given that a massive 40.3% of our samples originated from patients with unknown nationality, this investigation could not be realized with our data, but remains tempting to explore.

Moreover, the majority of patients (57.8%) from whom samples for the study were recovered were residents of the Emirate of Abu Dhabi, which also included the majority of participating centers (44.5%). Obviously, this conforms with the fact that Abu Dhabi was the first Emirate to start AMR surveillance, and it also is the largest Emirate in terms of area, where is occupies over 80% of the nation's land. However, Dubai, rather than Abu Dhabi, is the most populated Emirate, and samples from Dubai residents accounted for a much lower 24.1% only of those analyzed in this study. As such, these results must be cautiously interpreted.

As shown in Figure 3 and Supplementary Table 1, most recovered species were *E. faecalis* (81.5%), followed by *E. faecium* (8.5%). The remaining proportion was formed collectively from ten other species (4.8%) or has not been identified to the species level (5.3%). The species distribution resembles the historical situation in Europe three to four decades ago, when *E. faecalis* dominated all other species by far. Since then, in Europe *E. faecium* has gained a more important position within etiologically relevant enterococcal species, potentially due to the appearance of more virulent and/or environmentally stable strains (2, 49). Because of the more complex resistance pattern in *E. faecium*, this development has negative consequences in terms of efficient antibiotic therapy regimens.

Although there is frequent exchange of humans and goods between Europe and the UAE, the shift among enterococcal species has not been recorded in the latter, indicating the presence of local factors that stabilize the local species distribution among enterococci (48).

*Enterococcus* spp. strains were mostly associated with adults, while the percentage of isolates recovered from newborn and pediatric patients declined from 2010 to the end of the study period (Figure 4). As this finding has been observed similarly for several other pathogens under enhanced AMR surveillance in the UAE it is understood that this rather reflects a general demographic trend among the UAE (patient) population and is not particularly associated with *Enterococcus* infections.

Most of the *Enterococcus* spp. strains were isolated from urine (60.9%), followed by blood (6.0%), wound swabs (5.5%), and vaginal swabs (4.6%). In each case, the causative role of the isolates is debatable. In urine and vaginal swabs, enterococci represent parts of the physiological microflora, in most samples. In skin and intraabdominal wounds, enterococci again could be part of the local flora or, alternatively, could aggravate the situation in mixed species infections (86), but an independent causative role has not been demonstrated (87, 88). In many publications, the mere presence of enterococci in such wound samples is equated with a causative role (11), which is not acceptable in the light that the Koch postulates remain to be fulfilled for mixed species infections.

This differs from their responsibility in infections at normally sterile sites, such as endophthalmitis or periprosthetic infections—however, only a minority of isolates result from such sites in the present study. Still in blood cultures, enterococci could be contaminants from the skin microflora or could be involved in transient bacteremia as a result from intestinal translocation processes.

Without clinical details from the patients, neither the general number of isolates nor their association with specific materials necessarily reflect their etiological importance—a qualification that applies to all epidemiological studies on enterococci.

*Enterococcus* spp. was more frequently found in females (61.2%), as compared to males (38.8%), with a predominance of younger females (age 15–44), which was not equally observed in the male patient population (Figure 5). The predominance of younger females could be explained by the fact that urinary tract infections are more common in females than in males, and *Enterococcus* spp. is a common cause of urinary tract infections in the UAE, with 60.9% of *Enterococcus* spp. isolates being recovered from urinary tract samples (Figure 6). However, enterococcal urinary tract infections are frequently associated with inserted catheters. It is not clear whether the young female patients were more frequently subject to catheterization than other female age groups or males in general.

The proportion of outpatient samples was about 54%, while the remaining 46% of samples were recovered from inpatient settings, including 10.7% from ICU patients.

The evolution of antimicrobial resistance of *Enterococcus* species over the course of this study demonstrated that enterococci in the UAE show either high levels or increasing long-term trends (2010–2021) of acquired resistance to several clinically important antibiotic classes, in particular fluoroquinolones, aminoglycosides (HL) and glycopeptides.

Resistance of enterococci to fluoroquinolones (levofloxacin, moxifloxacin) was between 17 and 29% for *E. faecalis* and between 42 and 83% for *E. faecium*, with both showing a horizontal trend. National AMR surveillance data from a neighboring country (Oman) reported for 2018 a susceptibility level for ciprofloxacin of 34.1% (*E. faecalis*) and 17.4% (*E. faecium*), for blood isolates, but results need to be interpreted with caution due to low sample size (70). Such high level of resistance of enterococci to fluoroquinolones are a concern for the management of urinary tract infections (UTI), especially in the light of the fact that fluoroquinolones (mainly ciprofloxacin) are still the most prescribed empiric antibiotic for common urinary tract infections in the UAE, and despite that national guidelines have been published that do not recommend fluoroquinolones for the empiric treatment of urinary tract infections, due to the high fluoroquinolone resistance levels observed locally for common urinary tract pathogens (89).

Resistance of enterococci to high-level (HL) aminoglycosides has not been observed in the early years of AMR surveillance (2010–2015) and emerged in 2016. Overall, an increasing trend of resistance is observed for high-level gentamicin for *E. faecalis* (from 0% in 2013 to 12.5% in 2021; *p* < 0.001) and *E. faecium* (from 0% in 2010 to 12.6% in 2021; n.s.; Figure 7). Similarly, high-level resistance to streptomycin increased slightly for both pathogens, *E. faecalis* (2010: 0%, 2021: 2.5%; *p* < 0.001) and *E. faecium* (2010: 0%, 2021: 2.2%; *p* = 0.014). The molecular background for this development is unknown. However, it could be genetically linked to the vancomycin resistance phenotype, which would explain a similar increase over time. Enterococcal high level gentamicin resistance associated to vancomycin resistance has been noted elsewhere in Asia (90, 91).

This study demonstrates that vancomycin-resistant (VRE) and glycopeptide-resistant (GRE) enterococci are still relatively rare in the UAE, although slightly increasing over time in prevalence and relative importance. The relatively low numbers of VRE isolates found in this study could perhaps partially be explained by the fact that routine VRE screening procedures seem to be not as widely implemented among participating sites as compared to other MDRO-screening procedures, e.g., for MRSA, CRE, or, more recently, *Candida auris*.

While phenotypically vancomycin-resistant enterococci (VRE) were found in only 1.8% of *Enterococcus* spp. isolates overall, prevalence of VRE (%VRE) was highest for *E. faecium* (8.1%), followed by *E. faecalis* (0.9%). An increasing trend of resistance to glycopeptides (%VRE) has been observed for *E. faecalis* (2010: 0%, 2021: 0.6%; n.s.) and *E. faecium* (2010: 0%, 2021: 5.8%; n.s.). For *E. faecalis*, vancomycin-resistance was usually very low (< 1%), with a small peak in 2016 (2.2%). Teicoplanin showed similar resistance levels as compared to vancomycin, 0–1.7% (*E. faecalis*) and 0–12.4% (*E. faecium*), indicating the genomic presence of *vanA* as the responsible resistance genes in the majority of strains. Consecutively, resistance to teicoplanin followed the temporal trend already established for vancomycin resistance, i.e., from 0% (2010) to 0.6% (2021) for *E. faecalis* (n.s.), and from 0% (2010) to 4.3% (2021) for *E. faecium* (n.s.).

Lipopeptides (daptomycin) show an overall decreasing trend of resistance, from 3.8% (2013) to 1.4% (2021) for *E. faecalis* (*p* = 0.024) and from 25.0% (2016) to 2.6% (2021) for *E. faecium* (*p* = 0.026), which could be an attractive subject for further investigation as to elucidate the underlying mechanisms. Available national AMR surveillance data from other countries in the GCC region is scarce. Saudi Arabia reported for 2017 an average 90−92% susceptibility level to vancomycin for both, *E. faecalis* and *E. faecium*, with considerable regional variation (52−100%) (69), and Oman reported for 2018 susceptibility levels of 99.1% and 90.7% for *E. faecalis* and *E. faecium*, respectively (70).

Current resistance levels of enterococci in the UAE for oxazolidinones (linezolid), glycylglycines (tigecycline), and lipopeptides (daptomycin) are genetically not associated to the van-genes (92, 93) and thus, fortunately remain very low (linezolid, <2.4%; tigecycline, <2.8%), or are even decreasing (daptomycin), which still provides alternative treatment options for severe infections caused by enterococci (94). While this situation is better as compared to problems in the treatment of VRE strains elsewhere there is still a need to keep monitoring the situation to prevent future more virulent strains causing problems (95).

The percentages of MDR-*E. faecalis* and MDR-*E. faecium* increased during 2010–2021 (*p* < 0.001). A similar increase of the percentages of XDR-*E. faecalis* (*p* < 0.001) and XDR-*E. faecium* (n.s.) was observed. This indicates that there is a small but increasing fraction among the *E. faecium* VRE strains for which little to none therapeutic options are left. So far, none of the reporting hospitals signaled severe problems with such strains. Yet, the present analysis will lead to specific warning notices for hospitals in the UAE. In addition, antiseptic measures and decolonization strategies (96–98) will be considered for their integration into local hospital regimens.

As already discussed in the scientific community for enterococci in general, there is conflicting data on the role of VRE in severe infections concerning their contribution to increased mortality (99–102), possibly since in many studies the net effect of the underlying severe disease(s) are not sufficiently taken into consideration. However, there are potentially more tenacious and/or pathogenic VRE clones which remain for extended periods in specific hospitals and as a consequence, are involved in nosocomial outbreaks (38–40). Our data indicates that VRE infections are potentially associated with poor clinical outcome, in particular mortality rate, ICU admission rate, and excess hospitalization. The overall mortality rate, according to our observations, was about 3.0-fold higher in VRE-patients compared to those associated with non-VRE. In addition, we were able to demonstrate that patients associated with VRE were 2.2-fold more likely to be admitted to ICU, and their median length of stay was increased by 6 days, as compared to patients with non-VRE. This indicates a potential causative role and association with poor clinical outcomes, and is consistent with other findings that indicated high mortality rate and poor outcomes in patients with VRE (41, 103) but contradicts other studies that have not found such an association (12, 104, 105).

The collateral impact of the Coronavirus Disease 2019 (COVID-19) pandemic on AMR surveillance and stewardship, incidence of multidrug-resistant infections and antimicrobial resistance levels and trends has been subject to scientific debate (78–82). On one hand, surges in COVID-19 cases—and associated consequences like abandonment of antibiotic stewardship programs, high rates of antibiotic prescribing, and disorganization of patient care—were found to favor the spread of resistant bacteria. On the other hand, public health interventions implemented to control COVID-19—including patient lockdowns, universal masking, and reinforcement of hand hygiene—may provide the side-effect benefit of preventing bacterial transmission (78).

This study presents data from the UAE national AMR surveillance program, indicating a temporary negative impact of the COVID-19 early pandemic period (2020) on the total number of reported non-duplicate isolates/patients (all organisms), as compared to the pre-COVID-19 pandemic period (2010–2019), and 2021 (Supplementary Figure 2). The number of isolates reported for *Enterococcus* spp. (C) increased consistently during the whole study period (2010–2021), suggestive for an only minor impact of the COVID-19 pandemic on reporting rates for *Enterococcus* spp., including VRE.

Studies to date report heterogenous impacts of the pandemic on antibiotic-resistant bacteria. One review highlights a decreased incidence of healthcare associated infections caused by vancomycin-resistant enterococci (VRE) and methicillin-resistant *Staphylococcus aureus* (MRSA) relative to pre-pandemic levels (81). Yet in an analysis of microbiological data from 81 hospitals in the United States of America, infections due to MRSA, VRE, and multidrug-resistant gram-negative bacteria all spiked during local surges in COVID-19 cases (82). These conflicting reports suggest that impacts of COVID-19 on antibiotic resistance likely depend on the population, setting, and bacteria in question and may be highly context-specific (78).

This study presents data from the UAE, suggesting overall lower, or not further increasing, average levels of antibiotic resistance for *E. faecalis* and *E. faecium* against several clinically relevant antibiotics during the COVID-19 pandemic period (2020–2021), as compared to the pre-pandemic period (2010–2019; Figure 7, Table 1). *Enterococcus faecalis* showed a reduced average resistance level toward seven out of nine antibiotics (with the exception of HL-aminoglycosides) during the COVID-19 pandemic, as compared to the pre-COVID-19 period. *Enterococcus faecium* showed a reduced average resistance level toward six out of nine antibiotics (except for ampicillin, levofloxacin, and linezolid) during the COVID-19 pandemic, as compared to the pre-COVID-19 period.

## 5 Conclusion

Data are scarce in the UAE and whole MENA region for VRE-infections. Our data demonstrates that vancomycin-resistant (VRE) and glycopeptide-resistant (GRE) enterococci are relatively rare in the UAE, however, are showing a high, or increasing trend of resistance for several clinically important antibiotics classes, causing a concern for the treatment of serious infections caused by enterococci. This study also demonstrates that VRE are associated with higher mortality, increased ICU admission rates, and longer hospitalization, thus poorer clinical outcome, and higher associated costs in the UAE. We recommend the expansion of current surveillance techniques (e.g., local VRE screening), stricter infection prevention and control strategies, and better stewardship interventions. Further studies on the genetic and molecular epidemiology of enterococci are needed to characterize in more detail the clonal types circulating in the UAE, and their association with antimicrobial resistance, health outcome, and outbreaks of healthcare-associated infections.

## Data availability statement

The national AMR Surveillance database managed by the UAE Ministry of Health and Prevention (MOHAP) contains confidential health information, and as such can only be made available upon reasonable request from the UAE Ministry of Health and Prevention (https://mohap.gov.ae/).

## Ethics statement

Ethical approval for this study was provided by the Ministry of Health and Prevention Research Ethics Committee (MOHAP/DXB-REC/J.J.J./No. 86/2023), Dubai Scientific Research Ethics Committee (DSREC-GL17-2023), and Abu Dhabi Health Research and Technology Ethics Committee (DOH/ZHCD/2023/1316).

## Author contributions

JT: Conceptualization, Data curation, Formal analysis, Funding acquisition, Investigation, Methodology, Project administration, Resources, Software, Supervision, Validation, Visualization, Writing—original draft, Writing—review & editing. NA: Funding acquisition, Project administration, Supervision, Writing—review & editing. HA: Funding acquisition, Project administration, Supervision, Writing—review & editing. The UAE AMR Surveillance Consortium: Data curation, Investigation. GM: Conceptualization, Methodology, Supervision, Writing—review & editing. CM: Conceptualization, Methodology, Supervision, Writing—review & editing. DE: Conceptualization, Methodology, Supervision, Writing—review & editing. AS: Conceptualization, Methodology, Supervision, Writing—review & editing. AP: Conceptualization, Formal analysis, Methodology, Supervision, Writing—original draft.

## Figures and Tables

**Table:** 

**Nr**.	**Name**	**Institution**
1	Ahmed Elhag Ahmed	UAE University, College of Medicine and Health Sciences, Al Ain
2	Ahmed F. Yousef	Department of Biology, Center for Membranes and Advanced Water Technology, Khalifa University, Abu Dhabi
3	Amna AlBlooshi	Purelab, Al Ain
4	Dr. Adnan Alatoom	Sheikh Shakhbout Medical City (SSMC), Abu Dhabi
5	Dr. Ahmed Abdulkareem Al Hammadi	Tawam Hospital, Al Ain
6	Dr. Alaa MM Enshasy	Dubai Health Authority, Dubai
7	Dr. Amal Mubarak Madhi	Abu Dhabi Public Health Center, Abu Dhabi
8	Dr. Anju Nabi	Dubai Academic Health Corporation (DAHC), Dubai
9	Dr. Anup Shashikant Poddar	Al Sharq Hospital, Fujairah
10	Dr. Arun Kumar Jha	Danat Al Emarat Hospital, Abu Dhabi
11	Dr. Ayesha Abdulla Al Marzooqi	Abu Dhabi Public Health Center, Abu Dhabi
12	Dr. Bashir Aden	Khalifa University, Abu Dhabi
13	Dr. Deeba Jafri	Purelab, Sheikh Khalifa Medical City, Ajman
14	Dr. Duckjin Hong	Sheikh Khalifa Specialty Hospital (SKSH) RAK
15	Dr. Farah Ibrahim Al-Marzooq	United Arab Emirates University, Al Ain
16	Dr. Fatima Al Dhaheri	United Arab Emirates University, Al Ain
17	Dr. Ghada Abdel Wahab	Abu Dhabi Agriculture and Food Safety Authority, Abu Dhabi
18	Dr. Ghalia Abdul Khader Khoder	University of Sharjah, Sharjah
19	Dr. Gitanjali Avishkar Patil	NMC Specialty Hospital, Abu Dhabi
20	Dr. Hafiz Ahmad	RAK Hospital, Ras Al Khaimah
21	Dr. Hazim Khalifa	Department of Veterinary Medicine, UAE University, Al Ain
22	Dr. Husein Alzabi	Sheikh Khalifa General Hospital, Um al Quwain
23	Dr. Ibrahim Alsayed Mustafa Alhashami	Purelab, Al Qassimi Hospital, Sharjah
24	Dr. Irfaan Akthar	Mediclinic City Hospital, Dubai
25	Dr. Jens Thomsen	Abu Dhabi Public Health Center, Abu Dhabi
26	Dr. John Stelling	WHONET, Boston, USA
27	Dr. Kavita Diddi	Prime Hospital, Dubai
28	Dr. Krishnaprasad Ramabhadran	Burjeel Hospital, Abu Dhabi
29	Dr. Laila Al Dabal	Dubai Academic Health Corporation (DAHC, Dubai)
30	Dr. Madikay Senghore	Khalifa University, Abu Dhabi
31	Dr. Manal Abdel Fattah Ahmed	PureLab, Ras Al Khaimah
32	Dr. Maya Habous	Rashid Hospital, Dubai Academic Health Corporation, Dubai
33	Dr. Moeena Zain	American Hospital Dubai
34	Dr. Monika Maheshwari	Al Zahra Hospital, Dubai
35	Dr. Monika Maheshwari	Medeor 24x7 Hospital, Dubai
36	Dr. Mubarak Saif Alfaresi	Zayed Military Hospital, Abu Dhabi
37	Dr. Mushtaq Khan	United Arab Emirates University, Al Ain
38	Dr. Najiba Abdulrazzaq	Al Kuwait Hospital, Emirates Health Services Establishment, Dubai
39	Dr. Nehad Nabeel Al Shirawi	Al Fujairah Hospital
40	Dr. Nesrin Helmy	Mediclinic Al Noor Hospital - Khalifa Street, Abu Dhabi
41	Dr. Prashant Nasa	NMC Specialty Hospital Al Nahda, Dubai
42	Dr. Rajeshwari T. A. Patil	Burjeel Medical City, Abu Dhabi
43	Dr. Ratna A. Kurahatti	NMC Royal Hospital Khalifa City A, Abu Dhabi
44	Dr. Riyaz Amirali Husain	Dubai Hospital, Dubai Academic Health Corporation, Dubai
45	Dr. Robert Lodu Serafino Wani Swaka	Sheikh Shakhbout Medical City, Abu Dhabi
46	Dr. Savitha Mudalagiriyappa	University Hospital Sharjah, Sharjah
47	Dr. Seema Oommen	Burjeel Medical City, Abu Dhabi
48	Dr. Shaikha Ghannam Alkaabi	Abu Dhabi Public Health Center, Abu Dhabi
49	Dr. Simantini Jog	Fakeeh University Hospital, Dubai
50	Dr. Simantini Jog	King's College Hospital London Dubai Hills, Dubai
51	Dr. Siobhan O‘Sullivan	Khalifa University, Abu Dhabi
52	Dr. Somansu Basu	NMC Specialty Hospital, Al Ain
53	Dr. Yassir Mohammed Eltahir Ali	Animal Wealth Sector, Abu Dhabi Agriculture and Food Safety Authority, Abu Dhabi
54	Dr. Yousuf Mustafa Naqvi	Department of Health Abu Dhabi (DoH), Abu Dhabi
55	Dr. Zulfa Omar Al Deesi	Latifa Maternity & Pediatric Hospital, Dubai
56	Emmanuel Fru Nsutebu	Sheikh Shakhbout Medical City, Abu Dhabi
57	Fouzia Jabeen	Purelab, Sheikh Khalifa Hospital, Abu Dhabi
58	Francis Amirtharaj Selvaraj	Sheikh Khalifa Medical City (SKMC), Abu Dhabi
59	Hadayatullah Ghulam Muhammad	Emirates International Hospital, Al Ain
60	Imene Lazreg	University of Sharjah, Sharjah
61	Kaltham Ali Kayaf	Ministry of Climate Change & Environment (MOCCAE), Dubai
62	Laura Thomsen	University of Freiburg, Germany
63	Leili Chamani-Tabriz	Clemenceau Medical Center, Dubai
64	Pamela Fares Mrad	Abu Dhabi Public Health Center (ADPHC), Abu Dhabi
65	Pascal Frey	Berne University Hospital, Berne, Switzerland
66	Prof. Abiola Senok	College of Medicine, Mohammed Bin Rashid University of Medicine and Health Sciences, Dubai
67	Prof. Agnes-Sonnevend-Pal	University of Pécs, Pécs, Hungary
68	Prof. Andreas Podbielski	University Hospital Rostock, Rostock, Germany
69	Prof. Carole Ayoub Moubareck	College of Natural and Health Sciences, Zayed University, Dubai
70	Prof. Dean Everett	Department of Pathology and Infectious Diseases, College of Medicine, Khalifa University, Abu Dhabi
71	Prof. Godfred A. Menezes	Department of Medical Microbiology and Immunology, RAK Medical and Health Sciences University, Ras Al Khaimah
72	Prof. Hala Ahmed Fouad Ismail	PureLab, Al Qassimi Hospital, Sharjah
73	Prof. Mohamud M. Sheek-Hussein	United Arab Emirates University, Al Ain
74	Prof. Peter Nyasulu	Department of Global Health, Faculty of Medicine and Health Sciences, Stellenbosch University, South Africa
75	Prof. Sameh Soliman	University of Sharjah, Sharjah
76	Prof. Tibor Pal	University of Pécs, Pécs, Hungary
77	Rania El Lababidi	Dept. of Pharmacy Services, Cleveland Clinic Abu Dhabi
78	Saeed Hussein	Erada Center for Treatment and Rehabilitation, Dubai
79	Stefan Weber	Purelab, Abu Dhabi
80	Sura Khamees Majeed	Al Gharbia Hospitals - Madinat Zayed Hospital
81	Syed Irfan Hussein Rizvi	Mediclinic City Hospital, Dubai
82	Timothy Anthony Collyns	Tawam Hospital, Al Ain
83	Zahir Osman Babiker	Sheikh Shakhbout Medical City (SSMC), Abu Dhabi

## Supplementary material

Supplementary Figure 1 Geographical distribution of Enterococcus spp. non-duplicate isolates/patients over the surveillance period (2010–2021), by Emirate.

Supplementary Figure 2 Number of non-duplicate isolates/patients reported to National AMR Surveillance program, UAE, 2010–2021, by year.

Supplementary Figure 3 Length of inpatient hospitalization (days) for non-VRE patients **(A)** and VRE patients **(B)**, UAE, 2010–2021.

Supplementary Figure 4 Kaplan–Meyer curves for hospitalization duration (length of stay/LOS) of VRE patients vs. non-VRE patients, UAE, 2010–2021.

Supplementary Table 1 *Enterococcus* species distribution as overall percentages across the study period (UAE, 2010–2021).

## References

[1] Kåhrström CT Pariente N Weiss U. Intestinal microbiota in health and disease. Nature. (2016) 535:7610. 10.1038/535047a 27383978

[2] García-Solache M Rice LB. The enterococcus: a model of adaptability to its environment. Clin Microbiol Rev. (2019) 32:e00058-18. 10.1128/CMR.00058-18 30700430 PMC6431128

[3] Marutescu LG Popa M Gheorghe-Barbu I Barbu IC Rodríguez-Molina D Berglund F. Wastewater treatment plants, an “escape gate” for ESCAPE pathogens. Front Microbiol. (2023) 14:1193907. 10.3389/fmicb.2023.1193907 37293232 PMC10244645

[4] Werner G Abu Sin M Bahrs C Brogden S Feßler AT Hagel S. Therapierelevante Antibiotikaresistenzen im One-Health-Kontext [Therapy-relevant antibiotic resistances in a One Health context]. Bundesgesundheitsblatt Gesundheitsforschung Gesundheitsschutz. (2023) 66:628–43. 10.1007/s00103-023-03713-4 37184673 PMC10261244

[5] Munk P Brinch C Møller FD Petersen TN Hendriksen RS Seyfarth AM. Genomic analysis of sewage from 101 countries reveals global landscape of antimicrobial resistance. Nat Commun. (2022) 13:7251. 10.1038/s41467-022-34312-7 36456547 PMC9715550

[6] Kwit R Zajac M Smiałowska-Weglińska A Skarzyńska M Bomba A Lalak A. Prevalence of *Enterococcus* spp. and the whole-genome characteristics of *Enterococcus faecium* and *Enterococcus faecalis* strains isolated from free-living birds in Poland. Pathogens. (2023) 12:836. 10.3390/pathogens12060836 37375526 PMC10305306

[7] Zaheer R Cook SR Barbieri R Goji N Cameron A Petkau A. Surveillance of Enterococcus spp. reveals distinct species and antimicrobial resistance diversity across a One-Health continuum. Sci Rep. (2020) 10:3937. 10.1038/s41598-020-61002-5 32127598 PMC7054549

[8] Ali SA Bin-Asif H Hasan KA Rehman M Abbasi A. Molecular assessment of virulence determinants, hospital associated marker (IS16gene) and prevalence of antibiotic resistance in soil borne *Enterococcus* species. Microb Pathog. (2017) 105:298–306. 10.1016/j.micpath.2017.02.041 28258002

[9] Sanlibaba P Senturk E. Prevalence, characterization and antibiotic resistance of enterococci from traditional cheeses in Turkey. Int J Food Prop. (2018) 21:1955–63. 10.1080/10942912.2018.1489413

[10] Ferchichi M Sebei K Boukerb AM Karray-Bouraoui N Chevalier S Feuilloley MGJ. *Enterococcus* spp.: is it a bad choice for a good use-a conundrum to solve? Microorganisms. (2021) 9:2222. 10.3390/microorganisms9112222 34835352 PMC8622268

[11] Mehdorn M Kolbe-Busch S Lippmann N Moulla Y Scheuermann U Jansen-Winkeln B. Rectal colonization is predictive for surgical site infections with multidrug-resistant bacteria in abdominal surgery. Langenbecks Arch Surg. (2023) 408:230. 10.1007/s00423-023-02961-x 37301803 PMC10257639

[12] MacKenzie P Färber J Post M Esser T Bechmann L Kropf S. Previous antibiotic therapy as independent risk factor for the presence of vancomycin-resistant enterococci in surgical inpatients. Results from a matched case-control study. BMC Infect Dis. (2023) 23:274. 10.1186/s12879-023-08238-4 37131139 PMC10155433

[13] Bui MT Rohde AM Schwab F Märtin N Kipnis M Boldt A-C. Prevalence and risk factors of colonisation with vancomycin-resistant *Enterococci faecium* upon admission to Germany's largest university hospital. GMS Hyg Infect Control. (2021) 16:Doc06. 10.3205/dgkh000377 33643773 PMC7894188

[14] Grammatico-Guillon L Baron S Rosset P Gaborit C Bernard L Rusch E. Surgical site infection after primary hip and knee arthroplasty: a cohort study using a hospital database. Infect Control Hosp Epidemiol. (2015) 36:1198–207. 10.1017/ice.2015.148 26154882

[15] Cercenado E Torroba L Cantón R Martínez-Martínez L Chaves F García-Rodríguez JA. Multicenter study evaluating the role of enterococci in secondary bacterial peritonitis. J Clin Microbiol. (2010) 48:456–9. 10.1128/JCM.01782-09 19940047 PMC2815607

[16] Harbarth S Uckay I. Are there patients with peritonitis who require empiric therapy for enterococcus? Eur J Clin Microbiol Infect Dis. (2004) 23:73–7. 10.1007/s10096-003-1078-0 14735401

[17] Seguin P Brianchon C Launey Y Laviolle B Nesseler N Donnio P-Y. Are enterococci playing a role in postoperative peritonitis in critically ill patients? Eur J Clin Microbiol Infect Dis. (2012) 31:1479–85. 10.1007/s10096-011-1467-8 22076551

[18] Morvan AC Hengy B Garrouste-Orgeas M Ruckly S Forel JM Argaud L. Impact of species and antibiotic therapy of enterococcal peritonitis on 30-day mortality in critical care-an analysis of the OUTCOMEREA database. Crit Care. (2019) 23:307. 10.1186/s13054-019-2581-8 31492201 PMC6731585

[19] Pochhammer J Kramer A Schäffer M. Enterokokken und postoperative wundinfektionen: verursacher oder harmloser kommensale? [Enterococci and surgical site infections: causal agent or harmless commensals?]. Chirurg. (2017) 88:377–84. 10.1007/s00104-017-0388-1 28233041

[20] Fisher K Phillips C. The ecology, epidemiology and virulence of *Enterococcus*. Microbiology. (2009) 155(Pt 6):1749–57. 10.1099/mic.0.026385-0 19383684

[21] Tellapragada C Östlund H Giske C Rasmussen M Berge A. Recurrent bacteremia with *Enterococcus faecalis*, the clinical findings predicting endocarditis, and genomic characterization of the isolates: a retrospective cohort study. Eur J Clin Microbiol Infect Dis. (2023) 42:1001–9. 10.1007/s10096-023-04636-3 37422613 PMC10344971

[22] Djorić D Little JL Kristich CJ. Multiple low-reactivity class B penicillin-binding proteins are required for cephalosporin resistance in *Enterococci*. Antimicrob Agents Chemother. (2020) 64:e02273-19. 10.1128/AAC.02273-19 32041714 PMC7179317

[23] Kristich CJ Little JL. Mutations in the β subunit of RNA polymerase alter intrinsic cephalosporin resistance in Enterococci. Antimicrob Agents Chemother. (2012) 56:2022–7. 10.1128/AAC.06077-11 22290974 PMC3318385

[24] Lester CH Sandvang D Olsen SS Schønheyder HC Jarløv JO Bangsborg J. Emergence of ampicillin-resistant *Enterococcus faecium* in Danish hospitals. J Antimicrob Chemother. (2008) 62:1203–6. 10.1093/jac/dkn360 18765412

[25] Contreras GA Munita JM Arias CA. Novel strategies for the management of vancomycin-resistant enterococcal infections. Curr Infect Dis Rep. (2019) 21:22. 10.1007/s11908-019-0680-y 31119397

[26] Rafey A Nizamuddin S Qureshi W Anjum A Parveen A. Trends of vancomycin-resistant enterococcus infections in cancer patients. Cureus. (2022) 14:e31335. 10.7759/cureus.31335 36514590 PMC9741485

[27] Permana B Harris PNA Runnegar N Lindsay M Henderson BC Playford EG. Using genomics to investigate an outbreak of vancomycin-resistant *Enterococcus faecium* ST78 at a large tertiary hospital in Queensland. Microbiol Spectr. (2023) 11:e0420422. 10.1128/spectrum.04204-22 37191518 PMC10269735

[28] Islam M Sharon B Abaragu A Sistu H Akins RL Palmer K. Vancomycin resistance in *Enterococcus faecium* from the Dallas, Texas, area is conferred predominantly on pRUM-like plasmids. mSphere. (2023) 8:e0002423. 10.1128/msphere.00024-23 36939336 PMC10117061

[29] Palmer SM Ryback MJ. Vancomycin-resistant *Enterococci*. Pharmacotherapy. (1996) 16:819–29. 10.1002/j.1875-9114.1996.tb02999.x 8888077

[30] Leclercq R. Antibiotic resistance in streptococci and enterococci. Where are we, where are we going? An opening lecture. Adv Exp Med Biol. (1997) 418:419–27. 10.1007/978-1-4899-1825-3_101 9331685

[31] Arthur M Reynolds P. Courvalin, glycopeptide resistance in enterococci. Trends Microbiol. (1996) 4:401–7. 10.1016/0966-842X(96)10063-9 8899966

[32] Papadimitriou-Olivgeris M Filippidou S Kolonitsiou F Drougka E Koutsileou K Fligou F. Pitfalls in the identification of *Enterococcus* species and the detection of vanA and vanB genes. Lett Appl Microbiol. (2016) 63:189–95. 10.1111/lam.12610 27367648

[33] Li G Walker MJ De Oliveira DMP. Vancomycin resistance in *Enterococcus* and *Staphylococcus aureus*. Microorganisms. (2023) 11:24. 10.3390/microorganisms11010024 36677316 PMC9866002

[34] Prematunge C MacDougall C Johnstone J Adomako K Lam F Robertson J. VRE and VSE bacteremia outcomes in the era of effective VRE therapy: a systematic review and meta-analysis. Infect Control Hosp Epidemiol. (2016) 37:26–35. 10.1017/ice.2015.228 26434609 PMC4707508

[35] López-Luis BA Sifuentes-Osornio J Lambraño-Castillo D Ortiz-Brizuela E Ramírez-Fontes A Tovar-Calderón YE. Risk factors and outcomes associated with vancomycin-resistant *Enterococcus faecium* and ampicillin-resistant *Enterococcus faecalis* bacteraemia: a 10-year study in a tertiary-care centre in Mexico City. J Glob Antimicrob Resist. (2021) 24:198–204. 10.1016/j.jgar.2020.12.005 33359937

[36] Rottier WC Pinholt M van der Bij AK Arpi M Blank SN Nabuurs-Franssen MH. Attributable mortality of vancomycin resistance in ampicillin-resistant *Enterococcus faecium* bacteremia in Denmark and the Netherlands: a matched cohort study. Infect Control Hosp Epidemiol. (2022) 43:719–27. 10.1017/ice.2021.216 35670618

[37] Pinholt M Ostergaard C Arpi M Bruun NE Schønheyder HC Gradel KO. Incidence, clinical characteristics and 30-day mortality of enterococcal bacteraemia in Denmark 2006-2009: a population-based cohort study. Clin Microbiol Infect. (2014) 20:145–51. 10.1111/1469-0691.12236 23647880

[38] Piezzi V Wassilew N Atkinson A D'Incau S Kaspar T Seth-Smith HM. Nosocomial outbreak of vancomycin-resistant *Enterococcus faecium* (VRE) ST796, Switzerland, 2017 to 2020. Euro Surveill. (2020) 27:1–11. 10.2807/1560-7917.ES.2022.27.48.2200285 36695463 PMC9716646

[39] Kramer A Schwebke I Kampf G. How long do nosocomial pathogens persist on inanimate surfaces? A systematic review. BMC Infect Dis. (2006) 6:130. 10.1186/1471-2334-6-130 16914034 PMC1564025

[40] O'Driscoll T Crank CW. Vancomycin-resistant enterococcal infections: epidemiology, clinical manifestations, and optimal management. Infect Drug Resist. (2015) 24:217–30. 10.2147/IDR.S54125 26244026 PMC4521680

[41] Rödenbeck M Ayobami O Eckmanns T Pletz MW Bleidorn J Markwart R. Clinical epidemiology and case fatality due to antimicrobial resistance in Germany: a systematic review and meta-analysis, 1 January 2010 to 31 December 2021. Euro Surveill. (2023) 28:2200672. 10.2807/1560-7917.ES.2023.28.20.2200672 37199987 PMC10197495

[42] Arias CA Murray BE. The rise of the *Enterococcus*: beyond vancomycin resistance. Nat Rev Microbiol. (2012) 10:266–78. 10.1038/nrmicro2761 22421879 PMC3621121

[43] Poudel AN Zhu S Cooper N Little P Tarrant C Hickman M et al. The economic burden of antibiotic resistance: a systematic review and meta-analysis. PLoS ONE. (2023) 18:e0285170. 10.1371/journal.pone.0285170 37155660 PMC10166566

[44] van der Pol S Lokate M Postma MJ Friedrich AW. Costs of two vancomycin-resistant enterococci outbreaks in an academic hospital. Antimicrob Steward Healthc Epidemiol. (2023) 3:e8. 10.1017/ash.2022.365 36714289 PMC9879878

[45] Sugai M Yuasa A Miller RL Vasilopoulos V Kurosu H Taie A. An economic evaluation estimating the clinical and economic burden of increased vancomycin-resistant *Enterococcus faecium* infection incidence in Japan. Infect Dis Ther. (2023) 12:1695–713. 10.1007/s40121-023-00826-w 37302137 PMC10281932

[46] CDC. Vancomycin-resistant Enterococci (VRE) in Healthcare Settings. Centers for Disease Prevention and Control (2023). Available online at: https://www.cdc.gov/hai/organisms/vre/vre.html (accessed October 6, 2023).

[47] ECDC. Vancomycin-resistant Enterococci (VRE). European Centre for Disease Prevention and Control (2023). Available online at: https://www.ecdc.europa.eu/en/infectious-disease-topics/related-public-health-topics/antimicrobial-resistance/directory-guidance (accessed October 6, 2023).

[48] Pöntinen AK Top J Arredondo-Alonso S Tonkin-Hill G Freitas AR Novais C. Apparent nosocomial adaptation of *Enterococcus faecalis* predates the modern hospital era. Nat Commun. (2021) 12:1523. 10.1038/s41467-021-21749-5 33750782 PMC7943827

[49] Prieto AMG van Schaik W Rogers MRC Coque TM Baquero F Corander J. Global emergence and dissemination of enterococci as nosocomial pathogens: attack of the clones? Front Microbiol. (2016) 7:788. 10.3389/fmicb.2016.00788 27303380 PMC4880559

[50] Raven KE Reuter S Gouliouris T Reynolds R Russell JE Brown NM. Genome-based characterization of hospital-adapted *Enterococcus faecalis* lineages. Nat Microbiol. (2016) 1:15033. 10.1038/nmicrobiol.2015.33 27572164

[51] Gao W Howden BP Stinear TP. Evolution of virulence in *Enterococcus faecium*, a hospital-adapted opportunistic pathogen. Curr Opin Microbiol. (2018) 41:76–82. 10.1016/j.mib.2017.11.030 29227922

[52] Balkhy HH Zowawi H Albatshan HA Alshamrani MM Aidara-Kane A Erlacher-Vindel E. Antimicrobial resistance: a round table discussion on the “One Health” concept from the Gulf Cooperation Council Countries. Part one: a focus on leadership. J Infect Public Health. (2018) 11:771–7. 10.1016/j.jiph.2018.05.007 30396638

[53] Balkhy HH Zowawi HM Alshamrani MM Allegranzi B Srinivasan A Al-Abdely HM. Antimicrobial resistance: a round table discussion on the “One Health” concept from the Gulf Cooperation Council Countries. Part two: a focus on human health. J Infect Public Health. (2018) 11:778–83. 10.1016/j.jiph.2018.05.008 30396639

[54] Hannaoui I Barguigua A Serray B Mdaghri NE Timinouni M Chaoui AA. Intestinal carriage of vancomycin-resistant enterococci in a community setting in Casablanca, Morocco. J Glob Antimicrob Resist. (2016) 6:84–7. 10.1016/j.jgar.2016.03.008 27530846

[55] Benammar S Pantel A Aujoulat F Benmehidi M Courcol R Lavigne J-P. First molecular characterization of related cases of healthcare-associated infections involving multidrug-resistant *Enterococcus faecium* vanA in Algeria. Infect Drug Resist. (2018) 11:1483–90. 10.2147/IDR.S164487 30271181 PMC6149901

[56] Djahmi N Boutet-Dubois A Nedjai S Dekhil M Sotto A Lavigne JP. Molecular epidemiology of Enterococcus sp. isolated in a university hospital in Algeria. Scand J Infect Dis. (2012) 44:656–62. 10.3109/00365548.2012.673232 22568723

[57] Zerrouki H Rebiahi S-A Hadjadj L Ahlem F Elhabiri Y Sedrati T. High frequency and diversity of Vancomycin-resistant Enterococci (VRE) in Algerian healthcare settings. Infect Genet Evol. (2021) 92:104889. 10.1016/j.meegid.2021.104889 33933632

[58] Dziri R El Kara F Barguellil F Ouzari HI El Asli MS Klibi N. Vancomycin-resistant *Enterococcus faecium* in Tunisia: emergence of novel clones. Microb Drug Resist. (2019) 25:469–74. 10.1089/mdr.2018.0158 30403547

[59] Dziri R Lozano C Said LB Bellaaj R Boudabous A Slama KB. Multidrug-resistant enterococci in the hospital environment: detection of novel vancomycin-resistant *E. faecium* clone ST910. J Infect Dev Ctries 10. (2016) 799–806. 10.3855/jidc.8014 27580324

[60] Ahmed MO Elramalli AK Baptiste KE Daw MA Zorgani A Brouwer E. Whole genome sequence analysis of the first vancomycin-resistant *Enterococcus faecium* isolates from a Libyan Hospital in Tripoli. Microb Drug Resist. (2020) 26:1390–8. 10.1089/mdr.2019.0095 32181678

[61] Azzam A Elkafas H Khaled H Ashraf A Yousef M Elkashef AA. Prevalence of vancomycin-resistant enterococci (VRE) in Egypt (2010-2022): a systematic review and meta-analysis. J Egypt Public Health Association. (2023) 98:1–13. 10.1186/s42506-023-00133-9 37037955 PMC10086090

[62] Khairy RM Mahmoud MS Esmail MAM Gamil AN. First detection of vanB phenotype-vanA genotype vancomycin-resistant enterococci in Egypt. J Infect Dev Ctries. (2019) 13:837–42. 10.3855/jidc.10472 32074094

[63] Hashem YA Yassin AS Amin MA. Molecular characterization of *Enterococcus* spp. clinical isolates from Cairo, Egypt. Indian J Med Microbiol. (2015) 33(Suppl):80–6. 10.4103/0255-0857.148836 25657162

[64] Hassan RM Ghaith DM Ismail DK Zafer MM. Reduced susceptibility of *Enterococcus* spp. isolates from Cairo University Hospital to tigecycline: highlight on the influence of proton pump inhibitors. J Glob Antimicrob Resist. (2018) 12:68–72. 10.1016/j.jgar.2017.12.005 29274469

[65] Moemen D Tawfeek D Badawy W. Healthcare-associated vancomycin resistant *Enterococcus faecium* infections in the Mansoura University Hospitals intensive care units, Egypt. Braz J Microbiol. (2015) 46:777–83. 10.1590/S1517-838246320140403 26413060 PMC4568866

[66] Abdelkareem MZ Sayed M Hassuna NA Mahmoud MS Abdelwahab SF. Multi-drug-resistant *Enterococcus faecalis* among Egyptian patients with urinary tract infection. J Chemother. (2017) 29:74–82. 10.1080/1120009X.2016.1182358 27351108

[67] Ashgar AH. Frequency and antibiotic susceptibility of gram-positive bacteria in Makkah hospitals. Ann Saudi Med. (2011) 31:462–8. 10.4103/0256-4947.84622 21911982 PMC3183679

[68] Khalil MA Alorabi JA Al-Otaibi LM Ali SS Elsilk SE. Antibiotic resistance and biofilm formation in *Enterococcus* spp. isolated from urinary tract infections. Pathogens. (2022) 12:34. 10.3390/pathogens12010034 36678381 PMC9863506

[69] KSA MOH. Summary Report of Antibiogram Data from MOH Hospitals (2017). Riyadh: Ministry of Health, Saudi Arabia (2017).

[70] OMASS. Oman Antimicrobial Resistance Surveillance System. Annual Report 2018. Muscat: Sultanate of Oman Ministry of Health (2019).

[71] Ali GA Goravey W Najim MS Shunnar KM Ibrahim SI Daghfal J. Epidemiology, microbiological and clinical characteristics of *Enteroicoccus* species bloodstream infections: a 10-year retrospective cohort study from Qatar. Ann Med Surg. (2022) 80:104258. 10.1016/j.amsu.2022.104258 36045800 PMC9422275

[72] Sharaf EJ Senok AC Udo EE Botta GA. Trafficking of methicillin-resistant staphylococci and co-colonization with vancomycin-resistant enterococci. Med Princ Pract. (2011) 20:253–8. 10.1159/000323598 21454996

[73] Emaneini M Hosseinkhani F Jabalameli F Nasiri MJ Dadashi M Pouriran R. Prevalence of vancomycin-resistant *Enterococcus* in Iran: a systematic review and meta-analysis. Eur J Clin Microbiol Infect Dis. (2016) 35:1387–92. 10.1007/s10096-016-2702-0 27344575

[74] Thomsen J Abdulrazzaq N AlRand H The UAE AMR Surveillance Consortium. Surveillance of antimicrobial resistance in the United Emirates: the early implementation phase. Front Public Health. (2023) 11:1247627. 10.3389/fpubh.2023.1247627 38074700 PMC10704098

[75] CLSI. CLSI M100 ED33, 2023. Access our Free Resources: M100 and M60 Performance Standards for Antimicrobial and Antifungal Susceptibility Testing. Clinical and Laboratory Standards Institute (2023). Availaable online at: https://clsi.org/all-free-resources/ (accessed October 6, 2023).

[76] EUCAST. Clinical Breakpoints - Breakpints and Guidance. European Committee on Antimicrobial Susceptibility Testing (2023). Available online at: https://www.eucast.org/clinical_breakpoints/ (acessed July 17, 2023).

[77] Magiorakos A-P Burns K Rodríguez Baño J Borg M Daikos G Dumpis U. Multidrug-resistant, extensively drug-resistant and pandrug-resistant bacteria: an international expert proposal for interim standard definitions for acquired resistance. Clin Microbiol Infect. (2012) 18:268–81. 10.1111/j.1469-0691.2011.03570.x 21793988

[78] Smith DRM Shirreff G Temime L Opatowski L. Collateral impacts of pandemic COVID-19 drive the nosocomial spread of antibiotic resistance: a modelling study. PLoS Med. (2023) 20:e1004240. 10.1371/journal.pmed.1004240 37276186 PMC10241372

[79] Loyola-Cruz MÁ Gonzalez-Avila LU Martínez-Trejo A Saldaña-Padilla A Hernández-Cortez C Bello-López JM. ESKAPE and beyond: the burden of coinfections in the COVID-19 pandemic. Pathogens. (2023) 12:743. 10.3390/pathogens12050743 37242413 PMC10222376

[80] Segala FV Pafundi PC Masciocchi C Fiori B Taddei E Antenucci L. Incidence of bloodstream infections due to multidrug-resistant pathogens in ordinary wards and intensive care units before and during the COVID-19 pandemic: a real-life, retrospective observational study. Infection. (2023) 51:1061–9. 10.1007/s15010-023-02000-3 36867310 PMC9983510

[81] O'Toole RF. The interface between COVID-19 and bacterial healthcare-associated infections. Clin Microbiol Infect. (2021) 27:1772–6. 10.1016/j.cmi.2021.06.001 34111586 PMC8182977

[82] Baker MA Sands KE Huang SS Kleinman K Septimus EJ Varma N. The impact of coronavirus disease 2019 (COVID-19) on healthcare-associated infections. Clin Infect Dis. (2022) 74:1748–54. 10.1093/cid/ciab688 34370014 PMC8385925

[83] Global Media Insight (GMI). United Arab Emirates Population Statistics 2023. BLOG/infographics (2023). Available online at: https://www.globalmediainsight.com/blog/uae-population-statistics/ (accessed August 11, 2023).

[84] Vedadhir AA Rodrigues C Lambert H. Social science research contributions to antimicrobial resistance: protocol for a scoping review. Syst Rev. (2020) 9:24. 10.1186/s13643-020-1279-y 32024549 PMC7003437

[85] Minssen T Outterson K Rogers Van Katwyk S Batista PHD Chandler CIR Ciabuschi F. Social, cultural and economic aspects of antimicrobial resistance. Bull World Health Organ. (2020) 98:823–A. 10.2471/BLT.20.275875 33293739 PMC7716096

[86] Chong KKL Tay WH Janela B Yong AMH Liew TH Madden L. Enterococcus faecalis modulates immune activation and slows healing during wound infection. J Infect Dis. (2017) 216:1644–54. 10.1093/infdis/jix541 29045678 PMC5854026

[87] Sanders JM Tessier JM Sawyer R Dellinger EP Miller PR Namias N. Does isolation of enterococcus affect outcomes in intra-abdominal infections? Surg Infect. (2017) 18:879–85. 10.1089/sur.2017.121 28994635

[88] Röhrborn A Wacha H Schöffel U Billing A Aeberhard P Gebhard B. Coverage of enterococci in community acquired secondary peritonitis: results of a randomized trial. Surg Infect. (2000) 1:95–107. 10.1089/109629600321137 12594897

[89] MOHAP UTI. National Guidelines on the Empiric Antibiotic Treatment of Urinary Tract Infections. Dubai: Ministry of Health and Prevention (2021).

[90] Backiam ADS Duraisamy S Karuppaiya P Balakrishnan S Chandrasekaran B Kumarasamy A. Antibiotic susceptibility patterns and virulence-associated factors of vancomycin-resistant enterococcal isolates from tertiary care hospitals. Antibiotics. (2023) 12:981. 10.3390/antibiotics12060981 37370300 PMC10295198

[91] Lin P-Y Chan S-Y Stern A Chen P-H Yang H-C. Epidemiological profiles and pathogenicity of Vancomycin-resistant *Enterococcus faecium* clinical isolates in Taiwan. PeerJ. (2023) 11:e14859. 10.7717/peerj.14859 36855433 PMC9968458

[92] Sadowy E. Linezolid resistance genes and genetic elements enhancing their dissemination in enterococci and streptococci. Plasmid. (2018) 99:89–98. 10.1016/j.plasmid.2018.09.011 30253132

[93] Sengupta M Sarkar R Sarkar S Sengupta M Ghosh S Banerjee P. Vancomycin and linezolid-resistant enterococcus isolates from a tertiary care center in India. Diagnostics. (2023) 13:945. 10.3390/diagnostics13050945 36900089 PMC10001185

[94] Twilla JD Finch CK Usery JB Gelfand MS Hudson JQ Broyles JE. Vancomycin-resistant *Enterococcus* bacteremia: an evaluation of treatment with linezolid or daptomycin. J Hosp Med. (2012) 7:243–8. 10.1002/jhm.994 22076962

[95] Dadashi M Sharifian P Bostanshirin N Hajikhani B Bostanghadiri N Khosravi-Dehaghi N. The global prevalence of daptomycin, tigecycline, and linezolid-resistant *Enterococcus faecalis* and *Enterococcus faecium* strains from human clinical samples: a systematic review and meta-analysis. Front Med. (2021) 8:720647. 10.3389/fmed.2021.720647 34568377 PMC8460910

[96] Roson-Calero N Ballesté-Delpierre C Fernández J. Insights on current strategies to decolonize the gut from multidrug-resistant bacteria: pros and cons. Antibiotics. (2023) 12:1074. 10.3390/antibiotics12061074 37370393 PMC10295446

[97] Veriato TS Fontoura I Oliveira LD Raniero LJ Castilho ML. Nano-antibiotic based on silver nanoparticles functionalized to the vancomycin-cysteamine complex for treating *Staphylococcus aureus* and *Enterococcus faecalis*. Pharmacol Rep. (2023) 75:951–61. 10.1007/s43440-023-00491-3 37171518 PMC10176295

[98] Denkel LA Schwab F Clausmeyer J Behnke M Golembus J Wolke S. CLIP-ID study group. Central-line associated bloodstream infections in intensive care units before and after implementation of daily antiseptic bathing with chlorhexidine or octenidine: a *post-hoc* analysis of a cluster-randomised controlled trial. Antimicrob Resist Infect Control. (2023) 12:55. 10.1186/s13756-023-01260-w 37270604 PMC10239202

[99] DiazGranados CA Zimmer SM Klein M Jernigan JA. Comparison of mortality associated with vancomycin-resistant and vancomycin-susceptible enterococcal bloodstream infections: a meta-analysis. Clin Infect Dis. (2005) 41:327–33. 10.1086/430909 16007529

[100] Cho SY Lee DG Choi SM Kwon JC Kim SH Choi JK. Impact of vancomycin resistance on mortality in neutropenic patients with enterococcal bloodstream infection: a retrospective study. BMC Infect Dis. (2013) 13:504. 10.1186/1471-2334-13-504 24164924 PMC3870976

[101] Hansen SGK Roer L Karstensen KT Hoegh SV Hansen F Klein K. Vancomycin-sensitive *Enterococcus faecium* bacteraemia – hospital transmission and mortality in a Danish University Hospital. J Med Microbiol. (2023) 72:001731. 10.1099/jmm.0.001731 37436043

[102] Frickmann H Köller K Veil I Weise M Ludyga A Schwarz NG. On the role of enterococci in the bloodstream: results of a single-center, retrospective, observational study at a German University Hospital. Eur J Microbiol Immunol. (2017) 7:284–95. 10.1556/1886.2017.00030 29403657 PMC5793698

[103] Brinkwirth S Martins S Ayobami O Feig M Noll I Zacher B. Germany's burden of disease of bloodstream infections due to vancomycin-resistant *Enterococcus faecium* between 2015-2020. Microorganisms. (2022) 10:2273. 10.3390/microorganisms10112273 36422343 PMC9717732

[104] Vehreschild MJGT Haverkamp M Biehl LM Lemmen S Fätkenheuer G. Vancomycin-resistant enterococci (VRE): a reason to isolate? Infection. (2019) 47:7–11. 10.1007/s15010-018-1202-9 30178076

[105] Hemapanpairoa J Changpradub D Santimaleeworagun W. Clinical impact of vancomycin treatment in ampicillin-susceptible enterococci bloodstream infections. Antibiotics. (2022) 11:1698. 10.3390/antibiotics11121698 36551355 PMC9774542

