# Supplementary figures and images for: Epidemiology of vancomycin-resistant enterococci in the United Arab Emirates: a retrospective analysis of 12 years of national AMR surveillance data
Source: Front Public Health. 2023 Nov 27;11:1275778. doi: 10.3389/fpubh.2023.1275778 (PMC10715431; doi:10.3389/fpubh.2023.1275778)

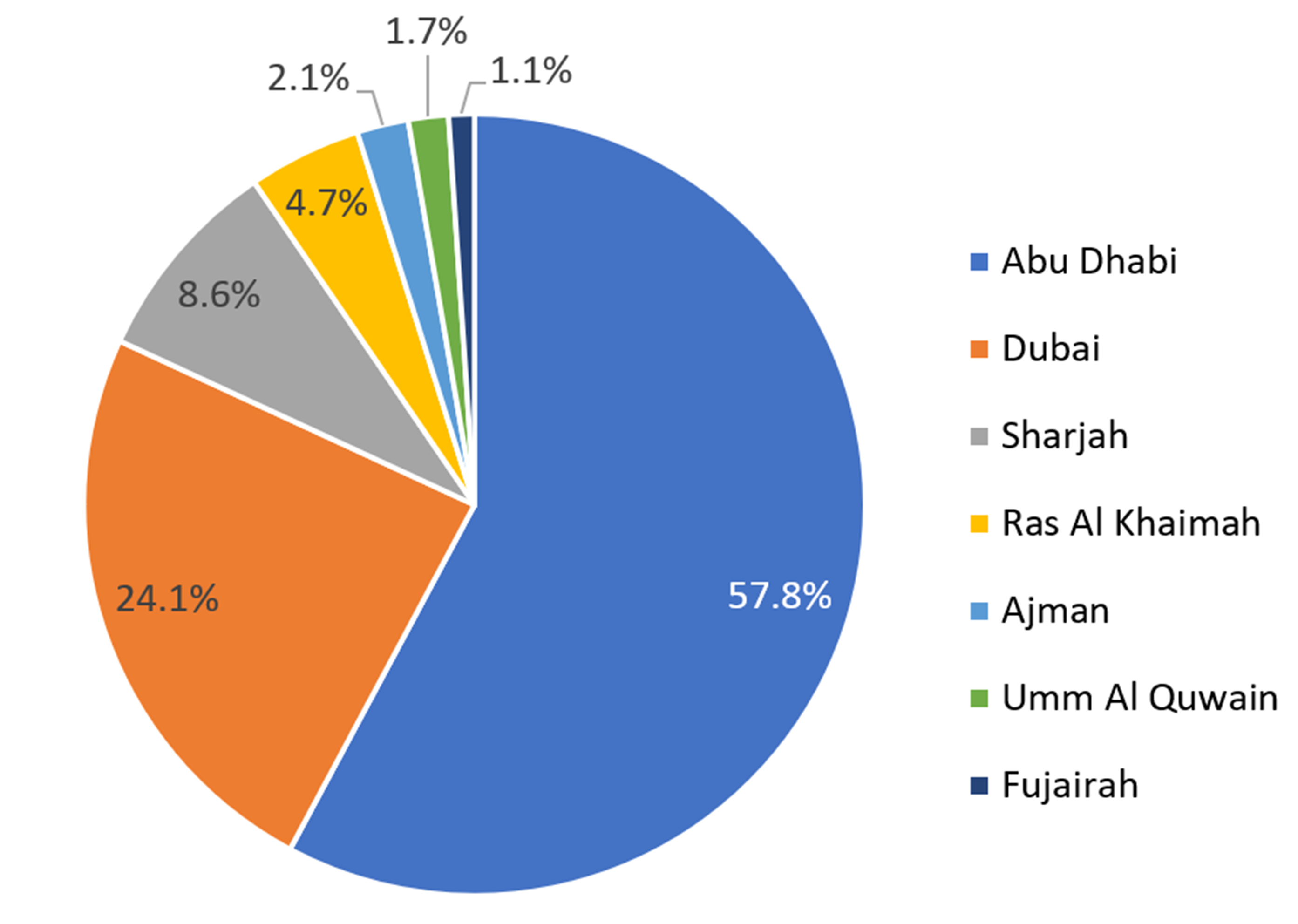

Supplement: Supplementary Figure 1 — Geographical distribution of Enterococcus spp. non-duplicate isolates/patients over the surveillance period (2010–2021), by Emirate. [file Image_1.tif]

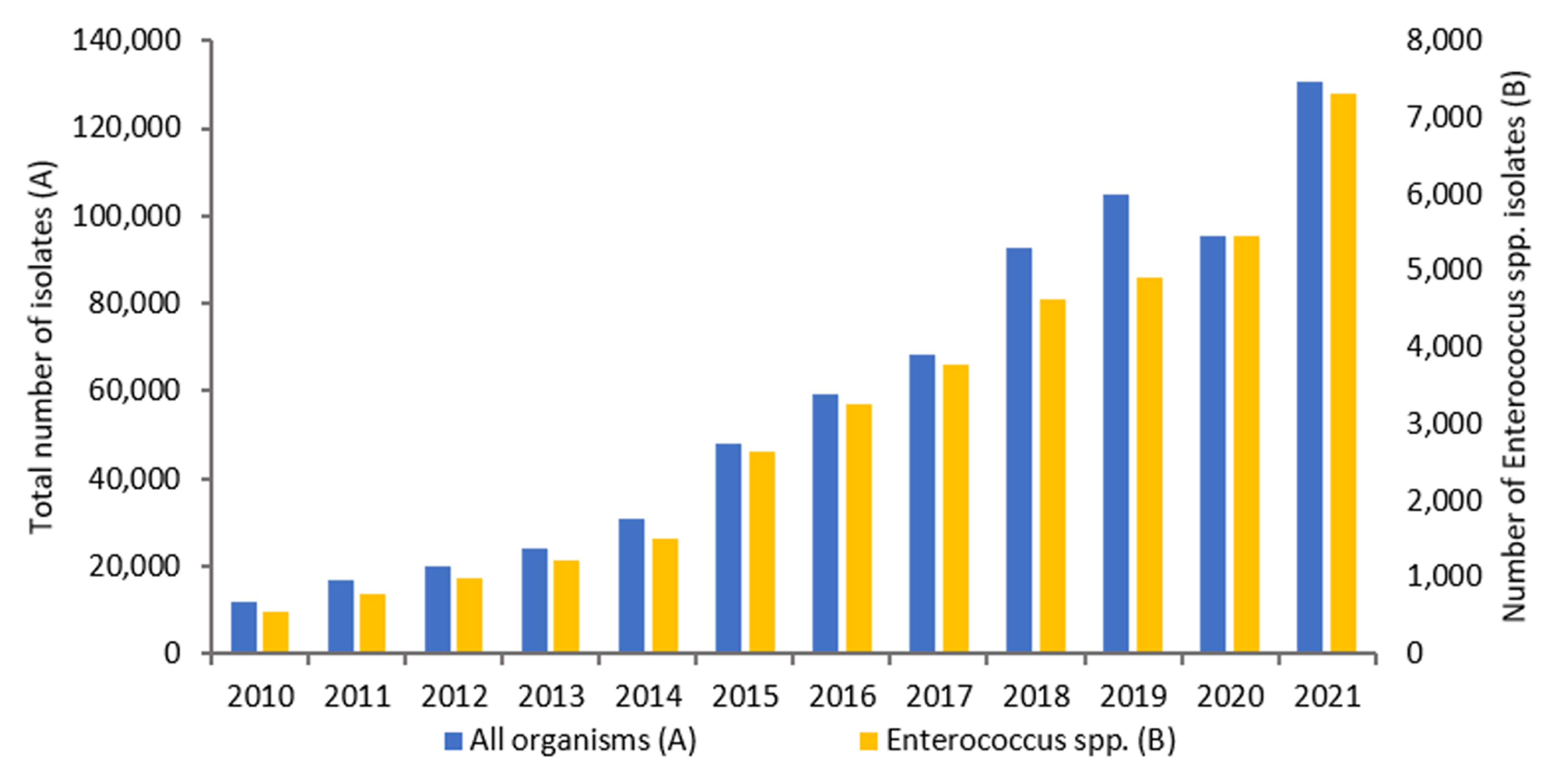

Supplement: Supplementary Figure 2 — Number of non-duplicate isolates/patients reported to National AMR Surveillance program, UAE, 2010–2021, by year. [file Image_2.TIF]

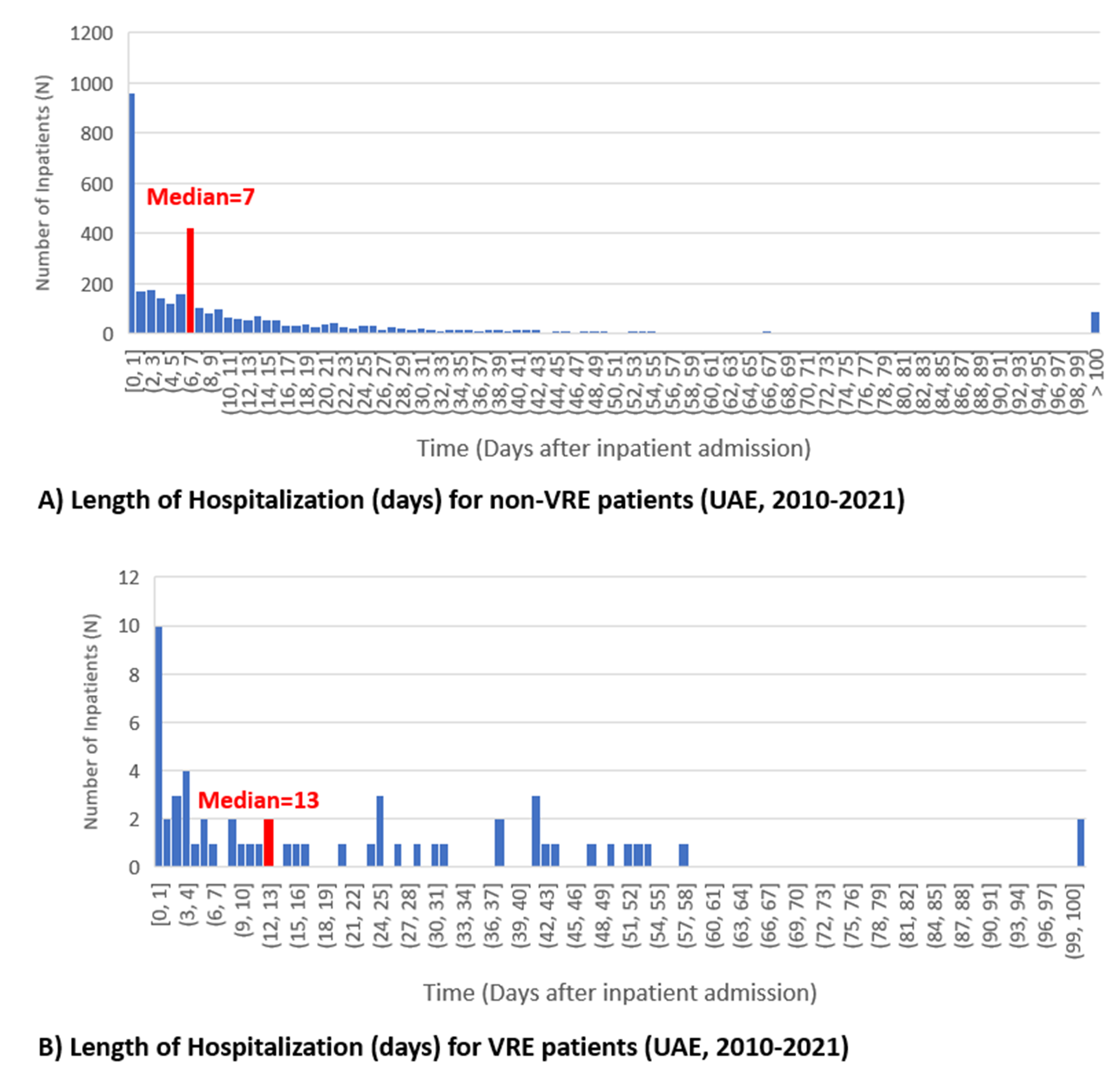

Supplement: Supplementary Figure 3 — Length of inpatient hospitalization (days) for non-VRE patients (A) and VRE patients (B), UAE, 2010–2021. [file Image_3.TIF]

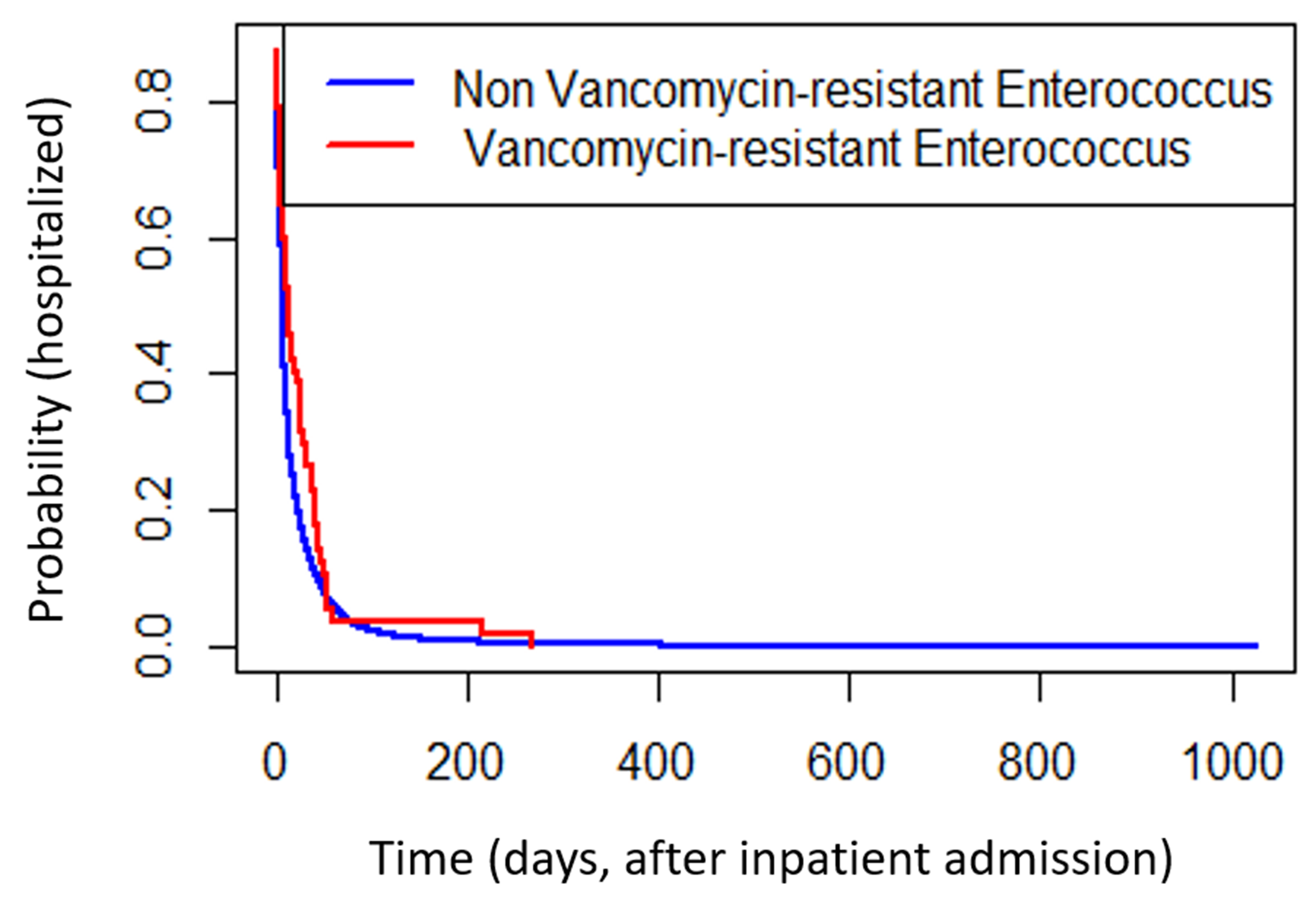

Supplement: Supplementary Figure 4 — Kaplan–Meyer curves for hospitalization duration (length of stay/LOS) of VRE patients vs. non-VRE patients, UAE, 2010–2021. [file Image_4.TIF]
