# Supplementary material for: Epidemiology of vancomycin-resistant enterococci in the United Arab Emirates: a retrospective analysis of 12 years of national AMR surveillance data
Source: Front Public Health. 2023 Nov 27;11:1275778. doi: 10.3389/fpubh.2023.1275778 (PMC10715431; doi:10.3389/fpubh.2023.1275778)
Supplement: Supplementary Table 1 — Enterococcus species distribution as overall percentages across the study period (UAE, 2010–2021). [file Table_1.pdf]

Supplementary Table 1. *Enterococcus* species distribution, as overall percentages across the study period (UAE, 2010-2021).

**Species name**	**Number (N)**	**Percentage (%)**
Enterococcus faecalis	30,893	81.5
Enterococcus faecium	3,226	8.5
Enterococcus avium	737	1.9
Enterococcus gallinarum	553	1.5
Enterococcus raffinosus	225	0.6
Enterococcus casseliflavus	137	0.4
Enterococcus hirae	85	0.2
Enterococcus durans	51	0.1
Enterococcus columbae	6	0.02
Enterococcus cecorum	4	0.01
Enterococcus malodoratus	1	0
Enterococcus saccharolyticus	1	0
Enterococcus sp.	1,990	5.3
Total	**37,909**	**100.0**
